# The RNA Binding Specificity of Human APOBEC3 Proteins Resembles That of HIV-1 Nucleocapsid

**DOI:** 10.1371/journal.ppat.1005833

**Published:** 2016-08-19

**Authors:** Ashley York, Sebla B. Kutluay, Manel Errando, Paul D. Bieniasz

**Affiliations:** 1 Laboratory of Retrovirology, The Rockefeller University, New York, New York, United States of America; 2 Aaron Diamond AIDS Research Center, The Rockefeller University, New York, New York, United States of America; 3 Department of Molecular Microbiology, Washington University School of Medicine in St. Louis, St. Louis, Missouri, United States of America; 4 Department of Physics, Washington University in St. Louis, St. Louis, Missouri, United States of America; 5 Howard Hughes Medical Institute, Aaron Diamond AIDS Research Center, New York, New York, United States of America; Duke University Medical Center, UNITED STATES

## Abstract

The APOBEC3 (A3) cytidine deaminases are antiretroviral proteins, whose targets include human immunodeficiency virus type-1 (HIV-1). Their incorporation into viral particles is critical for antiviral activity and is driven by interactions with the RNA molecules that are packaged into virions. However, it is unclear whether A3 proteins preferentially target RNA molecules that are destined to be packaged and if so, how. Using cross-linking immunoprecipitation sequencing (CLIP-seq), we determined the RNA binding preferences of the A3F, A3G and A3H proteins. We found that A3 proteins bind preferentially to RNA segments with particular properties, both in cells and in virions. Specifically, A3 proteins target RNA sequences that are G-rich and/or A-rich and are not scanned by ribosomes during translation. Comparative analyses of HIV-1 Gag, nucleocapsid (NC) and A3 RNA binding to HIV-1 RNA in cells and virions revealed the striking finding that A3 proteins partially mimic the RNA binding specificity of the HIV-1 NC protein. These findings suggest a model for A3 incorporation into HIV-1 virions in which an NC-like RNA binding specificity is determined by nucleotide composition rather than sequence. This model reconciles the promiscuity of A3 RNA binding that has been observed in previous studies with a presumed advantage that would accompany selective binding to RNAs that are destined to be packaged into virions.

## Author Summary

Cellular intrinsic immunity constitutes a key defense against infection by viruses. The APOBEC3 (A3) family of cytidine deaminases are intrinsic immune proteins that can hypermutate and destabilize retroviral genomes. For A3 proteins to exert their antiviral activity, they must be incorporated into nascent virions. Although A3 RNA binding activity has been shown to be critical for virion incorporation, the mechanism by which packaged RNA molecules are targeted by A3 proteins has been unclear. Therefore, we employed a cross-linking and deep sequencing strategy to analyze the targets of A3 proteins in HIV-1 infected cells and purified virions. We found that A3 proteins preferentially bind to particular types of RNA sequence in HIV-1 and cellular RNAs. In particular, we found that A3 proteins bind to G-rich and A-rich RNA sequences, a property that is reminiscent of that exhibited by the HIV-1 Gag protein. Further analyses revealed that A3 proteins preferentially bind the same sequences as the nucleocapsid domain of the HIV-1 Gag protein in viral RNA and cellular 7SL RNA. These results suggest a model of A3 incorporation into virions that that is based on mimicry of the RNA binding specificity of the HIV-1 nucleocapid protein.

## Introduction

APOBEC3 (A3) proteins are a family of germline-encoded proteins that inhibit the replication of a broad range of viruses and retroelements (reviewed in [1, 2]). A3 proteins exert their antiretroviral activity largely through their deoxycytosine deaminase activity, i.e. modification of dC-to-dU in single-stranded DNA retroviral reverse transcription intermediates, resulting in dG-to-dA hypermutation of the viral genome and error catastrophe [3–6]. In addition to inflicting genetic damage, alternative deamination-independent antiretroviral mechanisms have also been reported [7–11].

In primates, there are seven members of the A3 protein family that are categorized by their possession of either a single zinc (Z)-containing deaminase domain (A3A, A3C and A3H), or two Z domains (A3B, A3D, A3F and A3G) (reviewed in [12]). The antiviral activity of some A3 proteins is antagonized by most lentiviruses, including human immunodeficiency virus type-1 (HIV-1) through the action of the virion infectivity factor (Vif) protein. Vif targets A3 proteins for polyubiquitination and subsequent proteasomal degradation through the recruitment of core binding factor-β (CBF-β) and an E3 ubiquitin ligase complex, comprised of cullin 5, elongin B/C, and Rbx2 [13–19]. In the absence of a functional Vif protein, some A3 proteins are efficiently incorporated into progeny HIV-1 virions, enabling them to exert their antiviral effects during subsequent infection of a target cell.

Packaging of A3 proteins into HIV-1 virions depends on the nucleocapsid (NC) region of the viral Gag polyprotein and its associated RNA [20–25]. In the case of A3G, a large pocket within the A3G amino-terminal domain has been shown to contain residues that are critical for RNA binding, efficient particle incorporation and restriction [26–30]. These observations have led to the conclusion that A3 interacts with the NC region of Gag, indirectly, in an RNA-dependent manner for incorporation into virions.

Studies that have aimed to determine the identity of the RNA that is targeted by A3 proteins for incorporation into virions have yielded a variety of conclusions. One study indicated that viral RNA is targeted by A3 proteins [31], while another proposed that A3 proteins target 7SL [32], a cellular RNA that is normally part of the signal recognition particle (SRP) ribonucleoprotein complex but is enriched in retrovirus particles for unknown reasons [33–36]. Other reports have indicated that both cellular and viral RNA can be targeted by A3 proteins [21, 23]. This latter notion has been supported by a recent study in which incorporation of A3F and A3G into virions could be driven by diverse RNA molecules [37]. Overall, these studies lead to a model in which A3 proteins are promiscuous, non-specific RNA binding proteins and are able to efficiently infiltrate nascent HIV-1 virions by binding to unoccupied regions of nearly any RNA in an infected cell.

Notably, if it is true that A3-RNA interaction is completely non-selective, then cellular RNAs would compete with viral RNAs for A3 binding. Because cellular RNAs are present in large excess over HIV-1 RNAs in infected cells, their presence should inhibit A3 incorporation into HIV-1 virions to a significant degree. Indeed, such a scenario would require that A3 proteins be associated with a large fraction of the RNA molecules in the cell in order to be incorporated into a correspondingly large fraction of virions. While some RNA binding proteins associate with RNA in this completely non-specific manner, many other RNA binding proteins bind to their target RNA molecules through the recognition of specific RNA sequences and/or structures (reviewed in [38]), using discrete, structured RNA binding domains (reviewed in [39, 40]). The RNA binding activity of A3G has been mapped to a small number of amino acids [26–30], suggesting the existence of a discrete RNA binding site, and hinting at a degree of RNA binding selectivity. However, the structural basis for RNA recognition by A3 proteins remains undefined. These considerations make it intuitively surprising that A3 proteins would bind to their target RNA molecules without recognizing specific RNA sequences or elements to at least some degree. Moreover, it is unclear how potentially competitive Gag and NC RNA binding in cells and virions would affect A3 binding to viral and cellular RNAs during virion genesis.

For these reasons, we undertook a detailed study of the interactions between A3 proteins and RNA. Recent advances in ribonomic technologies, such as cross-linking immunoprecipitation coupled to next generation sequencing (CLIP-seq (reviewed in [41]) have enabled high resolution mapping of protein-RNA interactions. We employed CLIP-seq to determine how A3 proteins that have potent antiviral activity and a different complement of Z domains (A3F, A3G and A3H) interact with RNA in cells and virions. While we confirmed that A3 proteins bind to several classes of RNAs in infected and uninfected cells and virions, we found that HIV-1 RNA was bound preferentially over cellular RNA in infected cells. Notably, like HIV-1 Gag and NC, we found that A3 proteins target RNA sequences that are G-rich and A-rich. Comparative analyses of Gag, NC and A3 binding in cells and immature and mature virions revealed that A3 proteins target sequences that are also preferred binding sites for the NC protein in the viral genome and the cellular 7SL RNA. Thus, these data suggest a model in which A3 incorporation into HIV-1 virions is facilitated by its ability to preferentially bind G- and A- rich RNA sequences, partly mimicking properties of the NC domain of the HIV-1 Gag protein [42]. This model reconciles the apparent promiscuity of A3 RNA binding with its demonstrated ability to be incorporated into virions in the presence of a vast excess of potentially distracting cellular RNA molecules.

## Results

### CLIP-seq identifies HIV-1 RNA sequences preferentially targeted by A3F, A3G and A3H

**Fig 1:**
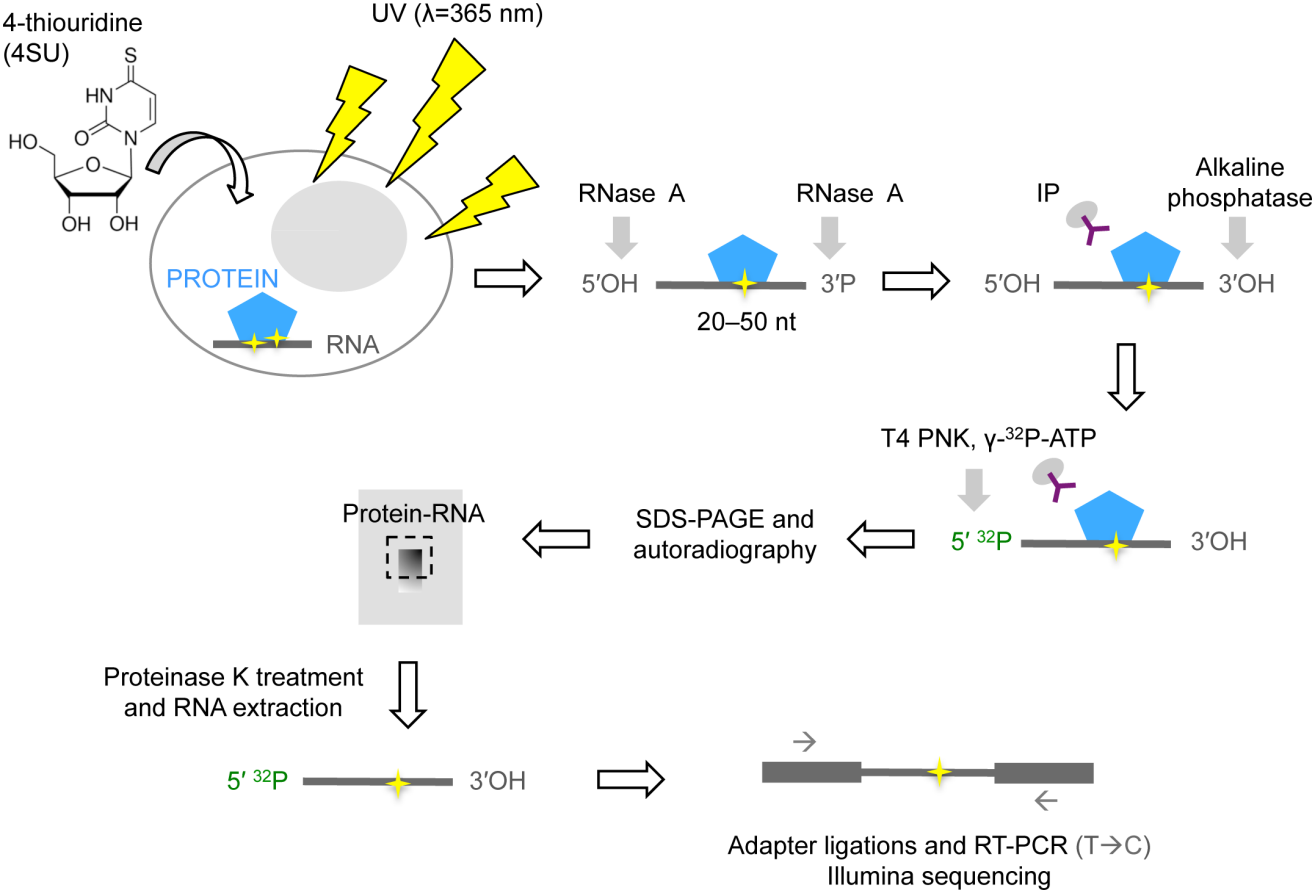
Flow diagram of CLIP-seq. Cells are fed with the ribonucleoside analog 4-thiouridine (4SU) which is incorporated into nascent RNA. Live cells are then irradiated with ultraviolet (UV) light, which induces covalent cross-links between proteins and RNA at sites of contact and 4SU incorporation. Cells are then lysed and treated with RNase A to generate oligonucleotides crosslinked to proteins of interest. Protein-RNA complexes are then immunopurified, and then the RNA is dephosphorylated at the 3'-end with alkaline phosphatase. The RNA is subsequently radiolabeled with ^32^P using T4 polynucleotide kinase (PNK) for detection by autoradiography. Protein-RNA complexes are then separated by SDS-PAGE, transferred to nitrocellulose and a region corresponding to protein-RNA adducts is excised. Cross-linked RNA is isolated by proteinase K treatment and phenol:chloroform extraction. After sequential adapter ligations, the RNA library is reverse transcribed. Reverse transcriptase often misincorporates a G opposite the 4SU cross-linking site, which leads to T to C substitutions in positive-strand of the cDNA, enabling the precise mapping of protein-RNA interaction sites. After PCR amplification, the cDNA library is sequenced by Illumina sequencing.

**Fig 2:**
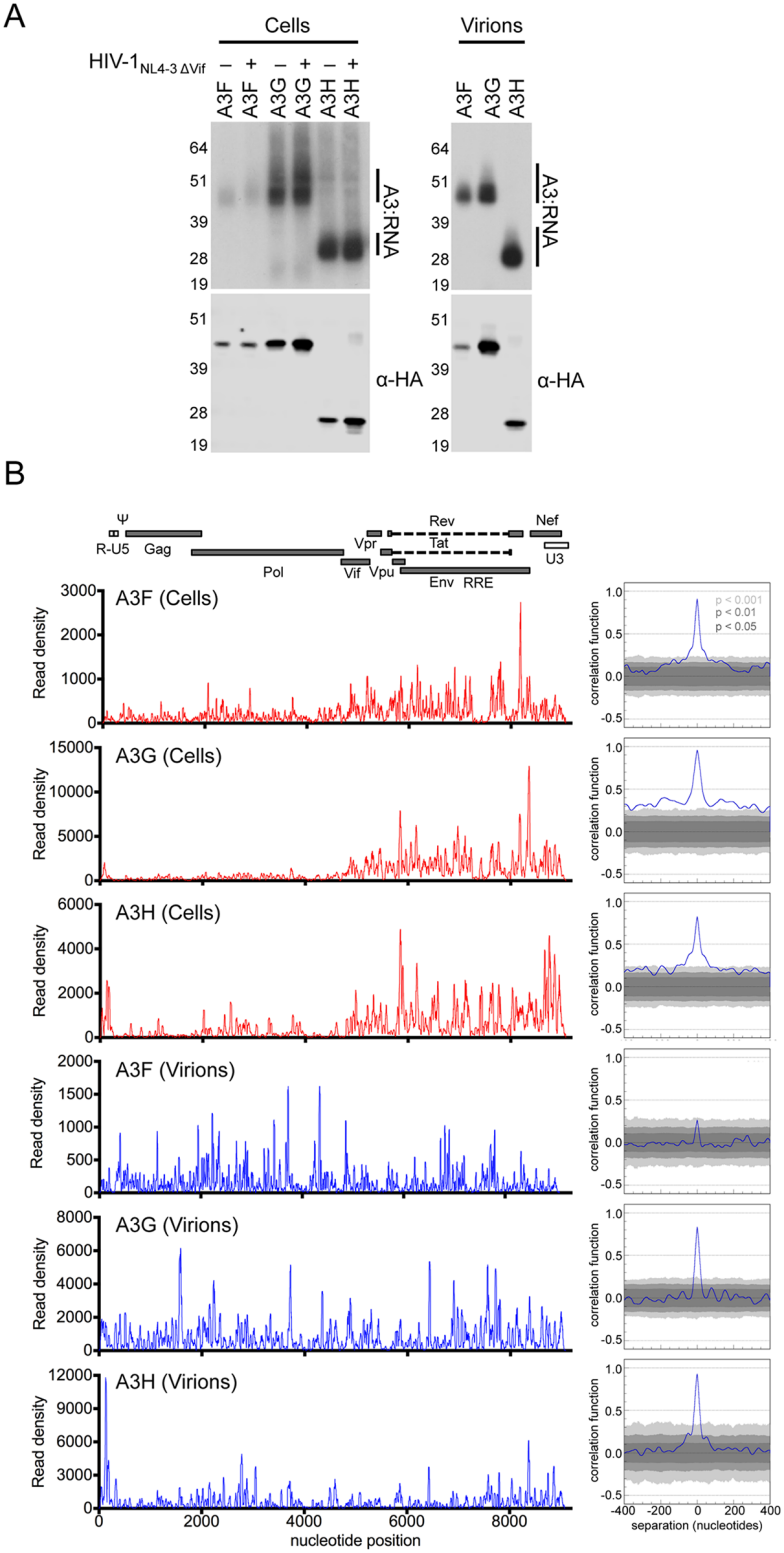
Viral RNA binding by A3F, A3G and A3H in cells and mature virions. (A) A3-RNA cross-linked complexes were immunoprecipitated from mock infected or HIV-1_NL4-3 ΔVif_ infected HEK 293T cells stably expressing 3×HA-tagged A3F, A3G or A3H proteins that had been fed with 4SU and UV-irradiated. A3-RNA cross-linked complexes were also immunoprecipitated from purified HIV-1_NL4-3 ΔVif_ virions that were derived from the 4SU-fed infected cells. Complexes were visualized by autoradiography (top) and Western blot analysis using a polyclonal anti-HA (bottom). (B) Frequency distribution of nucleotide occurrence (read density) in reads that were mapped to the HIV-1_NL4-3_ genome (left). A colinear schematic diagram of the HIV-1 genome is presented above. Correlation analyses (see methods) of A3 binding to viral RNA in cells from two independent CLIP experiments are shown (right).

We employed CLIP-seq techniques [42–44] to determine the RNA binding specificity of three unique members of the A3 protein family that exhibit potent anti-HIV-1 activity (A3F, A3G and A3H) (Fig 1). We first generated HEK 293T cell lines that stably express amino-terminally HA-tagged A3F and A3G and carboxyl-terminally HA-tagged A3H proteins. Then, cells were mock infected or infected with vesicular stomatitis virus G (VSV-G)-pseudotyped HIV-1_NL4-3 ΔVif_ and cultured in the presence of the ribonucleoside analog 4-thiouridine (4SU). Cells and purified virions were UV-irradiated, lysed, and digested with ribonuclease A. Thereafter, A3-RNA complexes were immunoprecipitated using an anti-HA antibody, 5'-end labeled with γ-^32^P-ATP and detected by autoradiography and Western blotting. As expected, A3F, A3G and A3H from cells and purified virions were cross-linked to RNA (Fig 2A). A3F was immunoprecipitated with reduced efficiency compared to A3G and A3H, resulting in fewer RNA molecules that were cross-linked to A3F for further analyses.

RNA oligonucleotides that were cross-linked to A3 were released by proteinase K digestion, purified, sequentially ligated to 5' and 3' adapters and converted to cDNA. After PCR amplification and next-generation sequencing of the resulting cDNA library, reads were subsequently mapped to the HIV-1 genome. The read density, a measure of the incidence of A3 binding to a specific RNA sequence on the HIV-1_NL4-3_ genome was determined by plotting the frequency with which each individual nucleotide in the viral genome was detected in mapped reads from CLIP libraries derived from cells and virions.

In cells, binding of A3F, A3G and A3H was observed at numerous sites throughout the viral genome. Discrete regions of the viral genome that yielded a high frequency of A3-bound reads were proximal to and interspersed with regions with low numbers of reads (Fig 2B and S1 Fig). The frequency of A3-binding events in the 3' half of the HIV-1_NL4-3_ RNA genome was higher than in the 5' half (Fig 2B). This finding potentially reflects the abundance of viral transcripts containing the 3' half of the viral genome, as it is represented in both spliced and unspliced viral mRNAs transcripts, while the 5' half is only present in unspliced transcripts. Correlation analyses revealed very clear reproducibility in the viral RNA sites occupied preferentially by each A3 protein in independent biological replicates, supporting the notion that A3 proteins bind preferentially to particular RNA sequences (Fig 2B and S1 Fig).

In virions, the read density peaks did not exhibit a bias towards the 3' half of the genome and instead were distributed across the entire length of the viral RNA (Fig 2B and S1 Fig). This likely reflects the uniform availability of viral sequences across the genome, as the full-length unspliced genomic RNA is selectively packaged into virions. As in cells, binding of A3F, A3G and A3H to viral RNA in mature virions reproducibly occurred at discrete sites (Fig 2B), with read density peaks containing high frequencies of reads interspersed with regions containing low frequencies of reads.

We were interested in how the RNA binding specificity of A3 proteins would be similar or different in a natural target of HIV-1, and so we performed A3F and A3G CLIP-seq experiments in MT4 cells as a representative T-cell line. Importantly, CLIP-seq experiments performed in a T- cell line (MT4) resulted in a similar distribution of binding sites on HIV-1 RNA indicating that cellular context does not greatly affect the HIV-1 RNA binding specificity of A3F and A3G (S2 Fig). Furthermore, using different crosslinking nucleotides (4SU or 6SG) resulted in a similar distribution of A3G and A3H binding sites on viral RNA (S3A and S3B Fig) indicating that the selective binding of A3 proteins to discrete sequence elements was not an artifact of the use of 4SU as the cross-linking nucleotide.

### Comparative analysis of A3F, A3G and A3H binding to HIV-1 RNA in cells and mature virions

**Fig 3:**
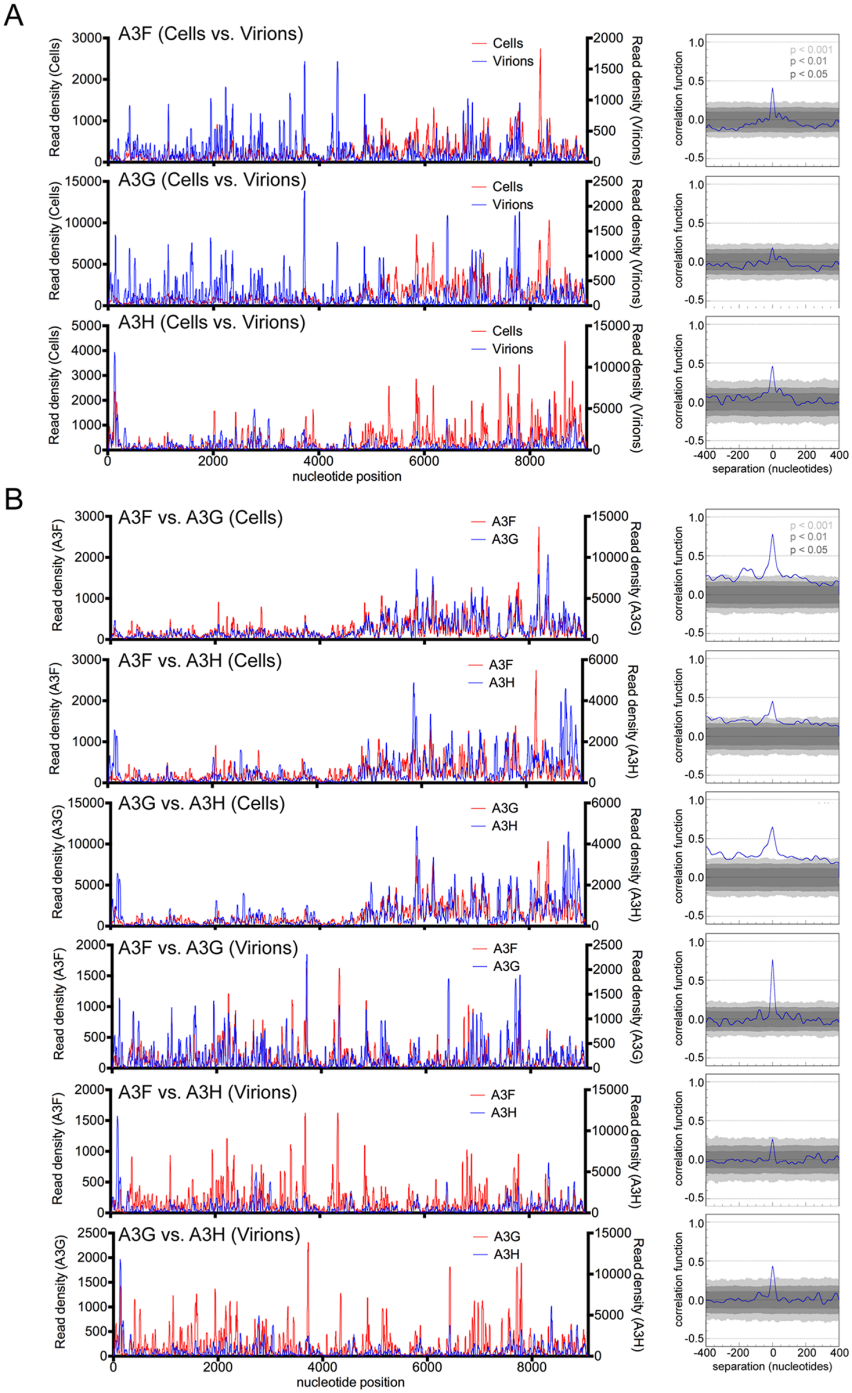
Comparison of A3F, A3G and A3H RNA binding in cells and mature virions. (A) Comparison of read density frequency distributions on the HIV-1_NL4-3_ genome for CLIP experiments in which the same A3 protein was immunoprecipitated from infected cells and mature virions (left). Correlation analyses of A3 binding frequencies across the viral RNA genome in infected cells and mature virions (right). (B) Comparisons of read density frequency distributions on the HIV-1_NL4-3_ genome for CLIP experiments in which different A3 proteins were immunoprecipitated from infected cells or mature virions (left). Correlation analyses of different A3 protein binding frequencies across the viral RNA genome in infected cells or mature virions (right).

To investigate potential similarities and differences in A3:RNA binding that might exist among the three A3 proteins in infected cells versus mature virions, we performed correlation analyses of RNA binding sites (Fig 3A and S4A Fig). Despite the fact that RNA sequences are differentially represented in cells versus virions, statistically significant correlation between RNA binding preferences in cells and mature virions was observed for each A3 protein, signifying that the overall binding specificity is consistent in the two environments, although some discrepancies were observed. The binding patterns of A3F, A3G and A3H to viral RNA revealed some differences in their intrinsic RNA binding specificities. For example, A3H had an increased preference for binding the R-U5 region of the viral RNA compared to A3F and A3G in cells (Fig 2B). Nevertheless, pair-wise comparisons of A3F, A3G and A3H binding sites in the HIV-1 genome in both cells and mature virions (Fig 3B and S4B Fig) showed statistically significant correlation of binding preferences among the A3 proteins. However, correlation functions for A3G or A3F versus A3H had less statistical support than did the A3F versus A3G comparison (Fig 3B). These findings imply that although the overall patterns of RNA binding of A3F, A3G and A3H are similar, A3H has subtle differences in RNA binding specificity compared to A3F and A3G, concordant with the fact that A3H (containing a single Z3 domain) is divergent from A3F and A3G (which contain Z1/Z1 and Z1/Z2 domains, respectively).

### A3 proteins preferentially bind to HIV-1 RNA over cellular mRNA

**Fig 4:**
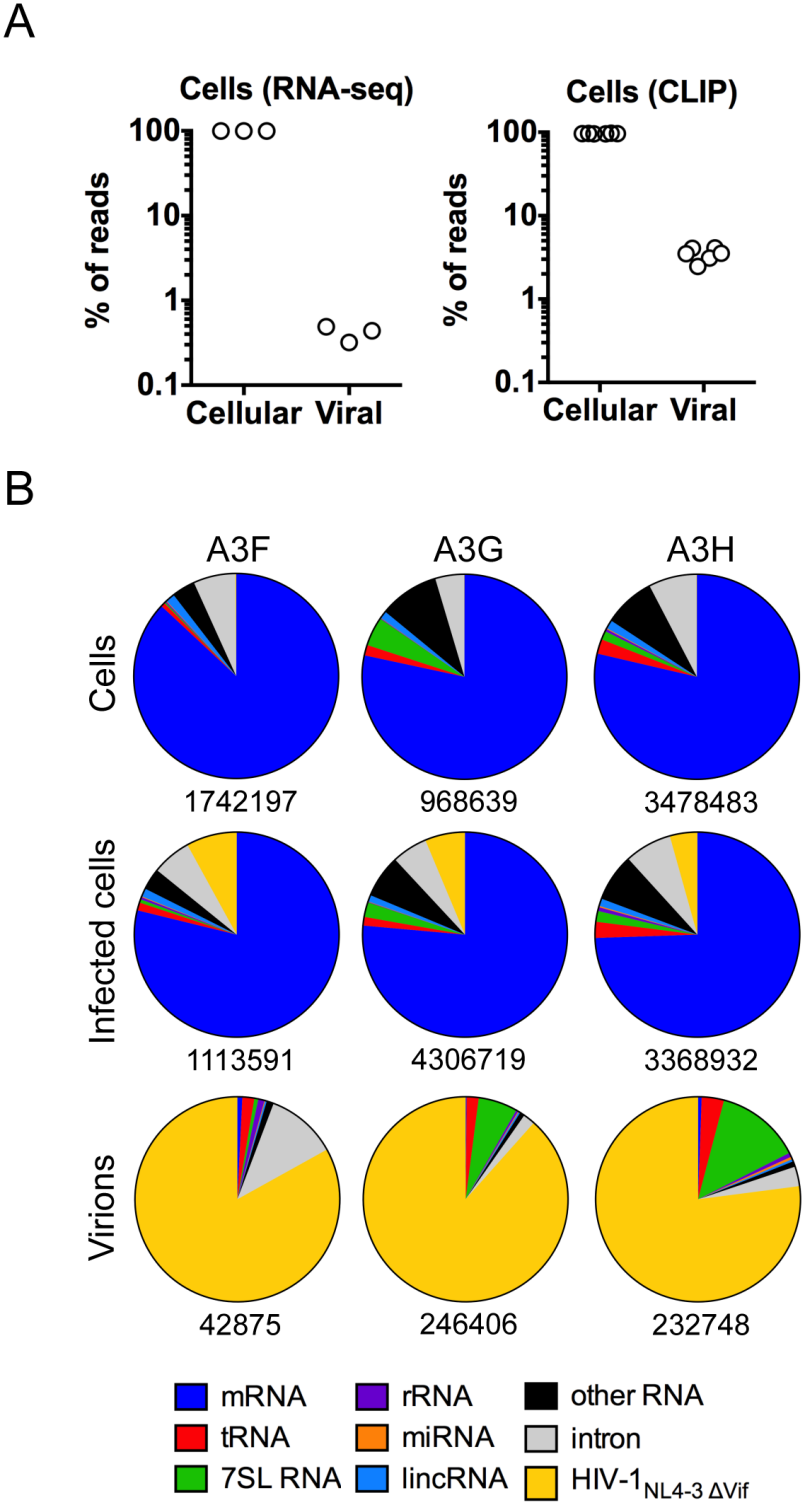
RNA targets of A3F, A3G and A3H in cells and mature virions. (A) Percentages of reads derived from the human and HIV-1 genomes in RNA-seq and A3-CLIP experiments performed using infected cells. Reads were normalized by cellular mRNA frequencies. (B) Origins of individual reads that map to the human and HIV-1_NL4-3_ genomes in A3 CLIP experiments with uninfected cells, infected cells and purified mature virions. Numbers below each chart are the total number of mapped reads.

While the A3 proteins bound to many sites on the viral genome, the aforementioned results suggested a degree of specificity in A3-RNA binding. To investigate RNA target specificity of A3 proteins we undertook a detailed inspection of RNA types and sequences that were most frequently bound by A3F, A3G and A3H in uninfected cells, infected cells and in purified virions. We aligned the raw reads derived from A3 CLIP-seq experiments done using infected cells to both the human and viral genomes and found that 2.5%–4.1% of the total reads were HIV-1 derived, whereas 97.5–95.9% were from cellular RNA (Fig 4A). In comparison, RNA sequencing (RNA-seq) libraries from identically infected cells contained only 0.3%–0.5% of reads derived from HIV-1, and ~99% were from cellular RNA (Fig 4A). Thus, viral RNA sequences appeared to be selectively bound (~5 to 12-fold more frequently than their occurrence) by A3 proteins in infected cells.

To investigate the basis for this apparent specificity, A3 CLIP reads were aligned to the human genome and the HIV-1 genome and analyzed using PARalyzer [45]. This approach defines a cluster, or preferred binding site, based on the incidence of a minimum number of overlapping reads that are proximal to dT-to-dC nucleotide substitutions that occur at the cross-linking site in 4SU-based CLIP assays. We counted the number of reads associated with each cluster, and each analysis was performed at least twice on independent biological replicate datasets. In uninfected cells, the majority of reads associated with clusters were mRNA-derived (~78–86%) with a minority of reads within clusters mapping to other cellular RNA types including tRNA, 7SL RNA, miRNA, and rRNA (Fig 4B). In HIV-1 infected cells, the majority of the cluster-associated reads were also messenger RNA (~74–78%) with reads derived from viral RNA representing ~4–8% of the total cluster-associated reads (Fig 4B). Indeed, viral RNA was the most frequently bound single RNA species in infected cells for each of the A3 proteins. In purified virions, the majority of cluster-associated reads were viral (~77–88%) with a minority of reads mapping to the cellular RNA that is present in HIV-1 virions (Fig 4B). In virions, 7SL RNAs represented 6.2% and 13.4% of reads bound by A3G and A3H respectively, whereas only 0.6% of the reads bound to A3F were from 7SL RNA in purified virions (Fig 4B). We also conducted CLIP-seq experiments using 6SG in A3G and A3H infected cells. This analysis resulted in a similar identification and classification of RNA binding sites, albeit with an even greater apparent selectivity of A3 proteins for viral RNA (S3C Fig). The greater number of G than U nucleotides in the HIV-1 genome may contribute to this slight discrepancy between 6SG and 4SU-based CLIP experiments.

As mRNAs constituted the preferred target of A3 proteins, compared to other types of cellular RNAs, we analyzed the binding pattern of A3 proteins to the 10 most frequently A3 bound mRNAs (S5, S6, S7 and S8 Figs). Comparisons of the read densities across the length of these mRNA sequences in the CLIP and RNA-seq experiments did not correlate, indicating that A3 proteins preferentially target specific portions of these transcripts, rather than binding evenly along the length of the transcript. Indeed, each of the A3 proteins exhibited a strong preference for binding to the 3'UTR sequences of these mRNAs, which likely reflects the lack of available sequences for binding in regions of the mRNAs that are actively translated by ribosomes (S5, S6, S7 and S8 Figs).

### A3 proteins preferentially bind G-rich and A-rich RNA sequences

These results clearly showed that A3 proteins have diverse RNA targets, but that binding is not indiscriminate. In cells, mRNAs represent the bulk of A3-bound RNA, with a clear preference for 3' untranslated sequences. Moreover, A3 proteins preferentially target viral RNA sequences in infected cells. These findings indicate that A3 proteins are incorporated into HIV-1 virions predominantly via interactions with viral RNA that are preferred over interactions with cellular mRNAs or other RNAs. However, it was unclear how viral RNA might be selected by A3 proteins from a diverse pool of RNAs in the cell. Note that some HIV-1 RNA species, including the full length packaged viral genome, have long 3'UTRs which might contribute to their apparent selection for binding by the A3 proteins (Fig 4A and 4B). An additional possibility was that A3 proteins might have selectivity for certain nucleotides or motifs, that occur with increased frequency in viral RNAs.

**Fig 5:**
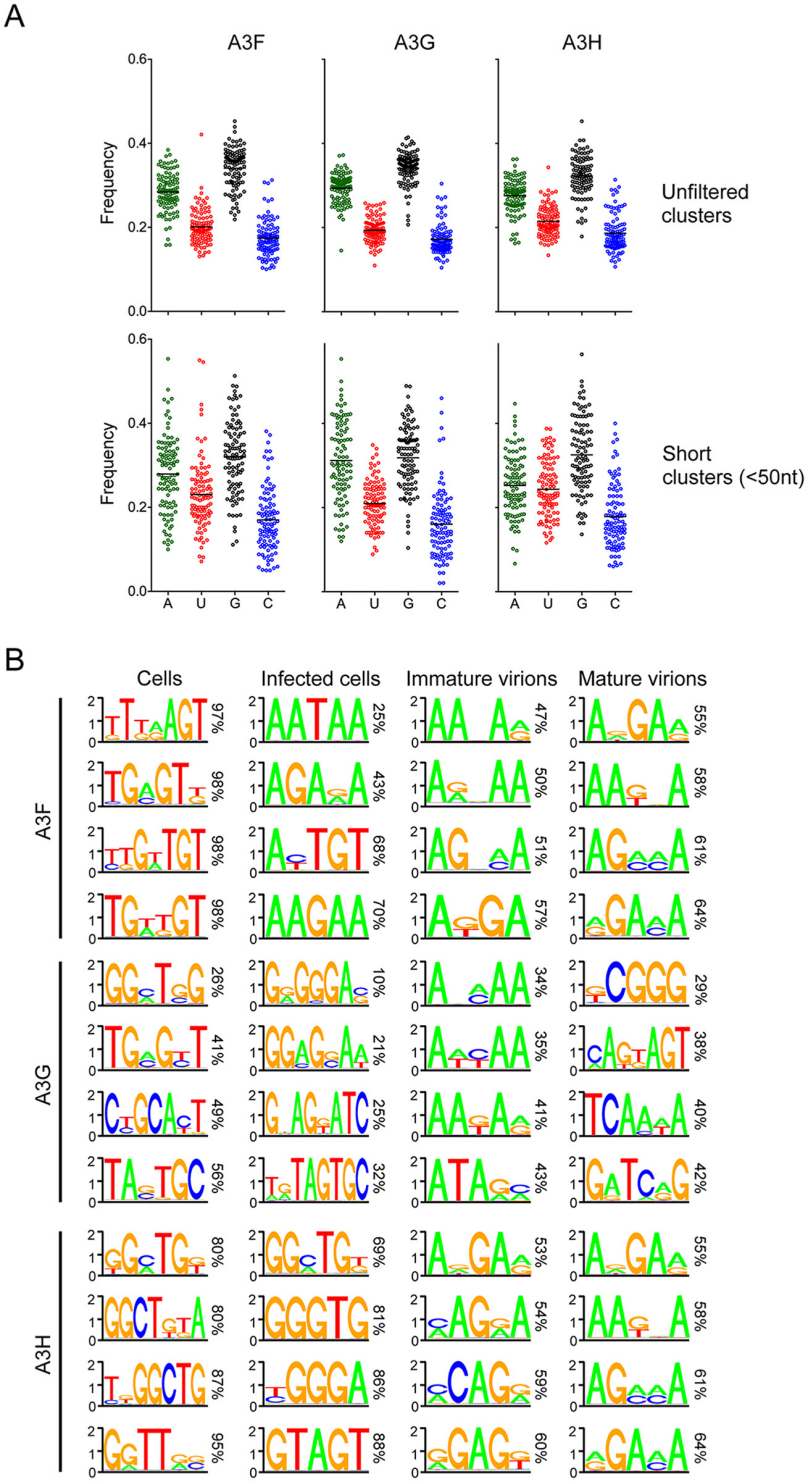
Nucleotide composition of preferred A3 protein binding sites. (A) The 100 PARalyzer-generated clusters in mRNAs with the greatest number of total associated reads in A3 CLIP experiments were inspected and the fraction the sequence composed of each of the four nucleotides was plotted (top). PARalyzer-generated clusters were filtered according to size (<50 nucleotides) and the clusters with the greatest number of total associated reads in A3 CLIP experiments were inspected and the fraction the sequence composed of each of the four nucleotides was plotted (bottom). (B) Deoxyribonucleotide sequence motifs identified by cERMIT in CLIP-seq cDNA libraries as most frequently present in A3-bound RNA in uninfected cells, infected cells and in immature and mature purified virions. Cumulative percentages of clusters containing the motifs are indicated.

To investigate this possibility, we isolated the 100 sites (clusters) in a complex RNA source (cellular mRNAs) that were most frequently bound by A3F, A3G and A3H in cells. This analysis selected clusters representing RNA regions of 200–900 nucleotides in length. Thus, they likely represent concentrations of several A3 protein binding sites rather than individual A3 binding sites. Next, we determined the nucleotide composition of these clusters, or collections of binding sites. This analysis revealed that the clusters had a striking propensity to be G-rich and/or A- rich (mean G-, A-, C- and U- content of ~35%, ~28%, ~17% and ~20%, respectively) compared to mean G-, A-, C- and U- content of ~24%, ~36%, ~18% and ~22%, of the HIV-1_NL4-3_ genome, and ~23%, ~25%, ~22% and ~30%, respectively, for human RNA as determined by our RNA-seq analysis) (Fig 5A). We also determined the nucleotide composition of short clusters (<50 nucleotides) where A3 protein binding had most frequently occurred, and were more likely to represent small numbers, or individual A3 binding sites. Similarly, in the case of A3F and A3G, we found these clusters to be G- and A-rich (mean G-, A-, C- and U- content of ~31%, ~30%, ~16% and ~23%, respectively), while A3H bound clusters were G-rich, but not as A-rich as A3F or A3G bound clusters (mean G-, A-, C- and U- content of ~32%, ~25%, ~18% and ~24%, respectively) (Fig 5A).

Additionally, we used cERMIT [46] to examine sequence motifs that occurred most frequently in A3-bound RNA clusters in uninfected cells, infected cells and immature and mature purified virions (Fig 5B). In this analysis, G-rich sequence motifs were most often identified in A3-bound clusters in uninfected cells. Conversely, in infected cells, the most frequently identified motifs in A3F and A3G bound RNAs were A-rich in addition to being G-rich. The unusual A-rich nature of the HIV-1 genome, and the apparent selectivity of A3 proteins for viral RNA could be responsible for this effect. However, motifs in A3H bound RNAs were mostly G-rich in both infected and uninfected cells. This finding along with the finding that short A3H binding clusters had a higher propensity to be G-rich rather than A-rich suggests that A3H might have a higher preference for G-rich rather than A-rich RNA as compared to A3F and A3G. Nevertheless, in purified immature and mature virions, A3-bound clusters were found to be G/A-rich, a result that is undoubtedly influenced by the dominance of the A-rich viral RNA in virions. Preferred A3 protein binding sites were correspondingly U- and C-poor, implying that G/A-rich sequences overall represent preferred binding sites for A3 proteins in infected cells.

### Comparative analysis of A3, Gag and NC binding to viral RNA during HIV-1 assembly

**Fig 6:**
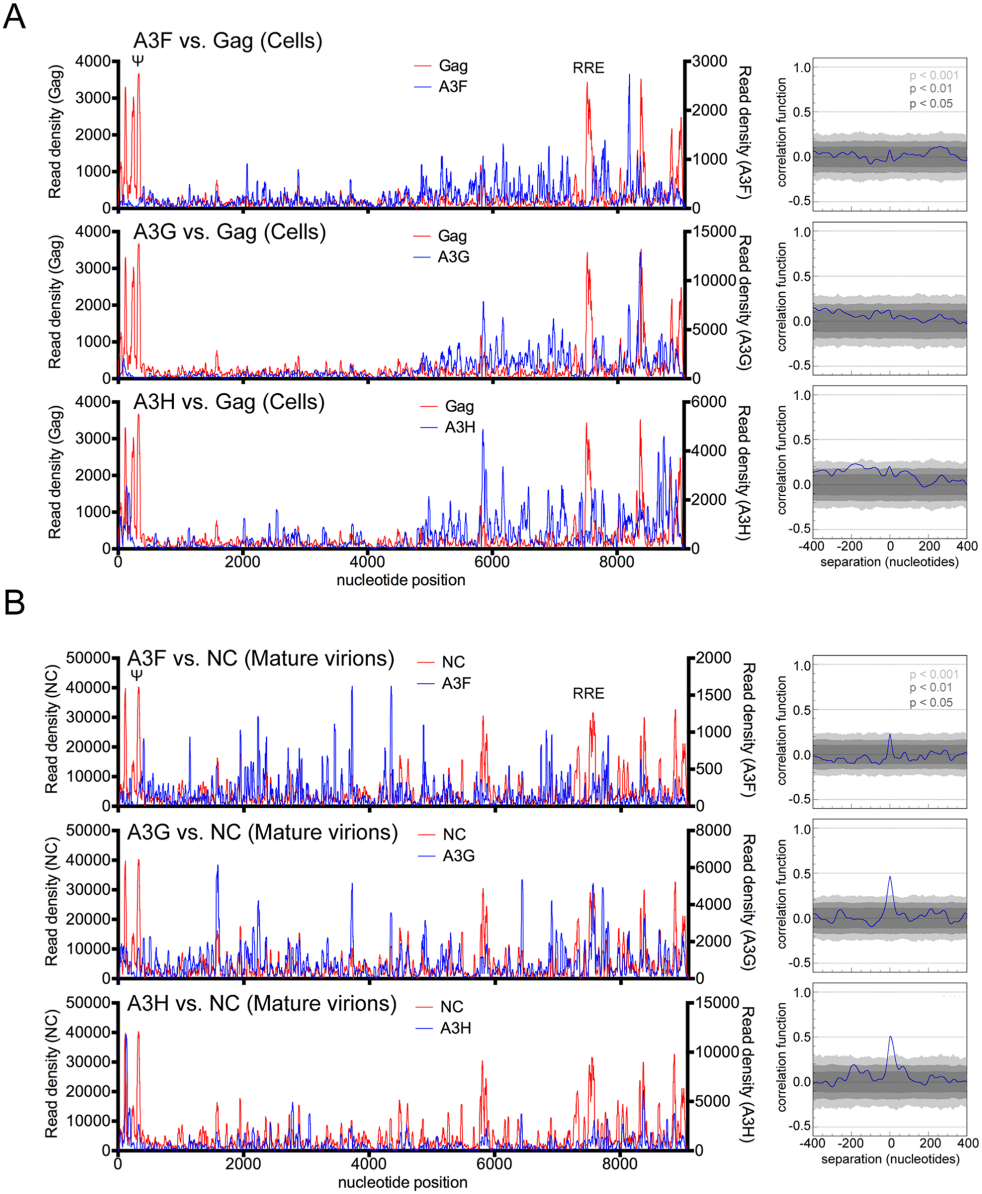
Interplay between A3, Gag and NC binding to HIV-1 RNA in infected cells or mature virions. (A) Comparisons of read density frequency distribution on the HIV-1_NL4-3_ genome for CLIP experiments in which A3 or Gag were immunoprecipitated from infected cells (left). Correlation analyses of A3 and Gag binding frequencies across the viral RNA genome in infected cells (right). (B) Comparisons of read density frequency distributions on the HIV-1_NL4-3_ genome for CLIP experiments in which A3 and NC were immunoprecipitated from purified mature virions (left). Correlation analyses of A3 and NC binding frequencies across the viral RNA genome (right).

**Fig 7:**
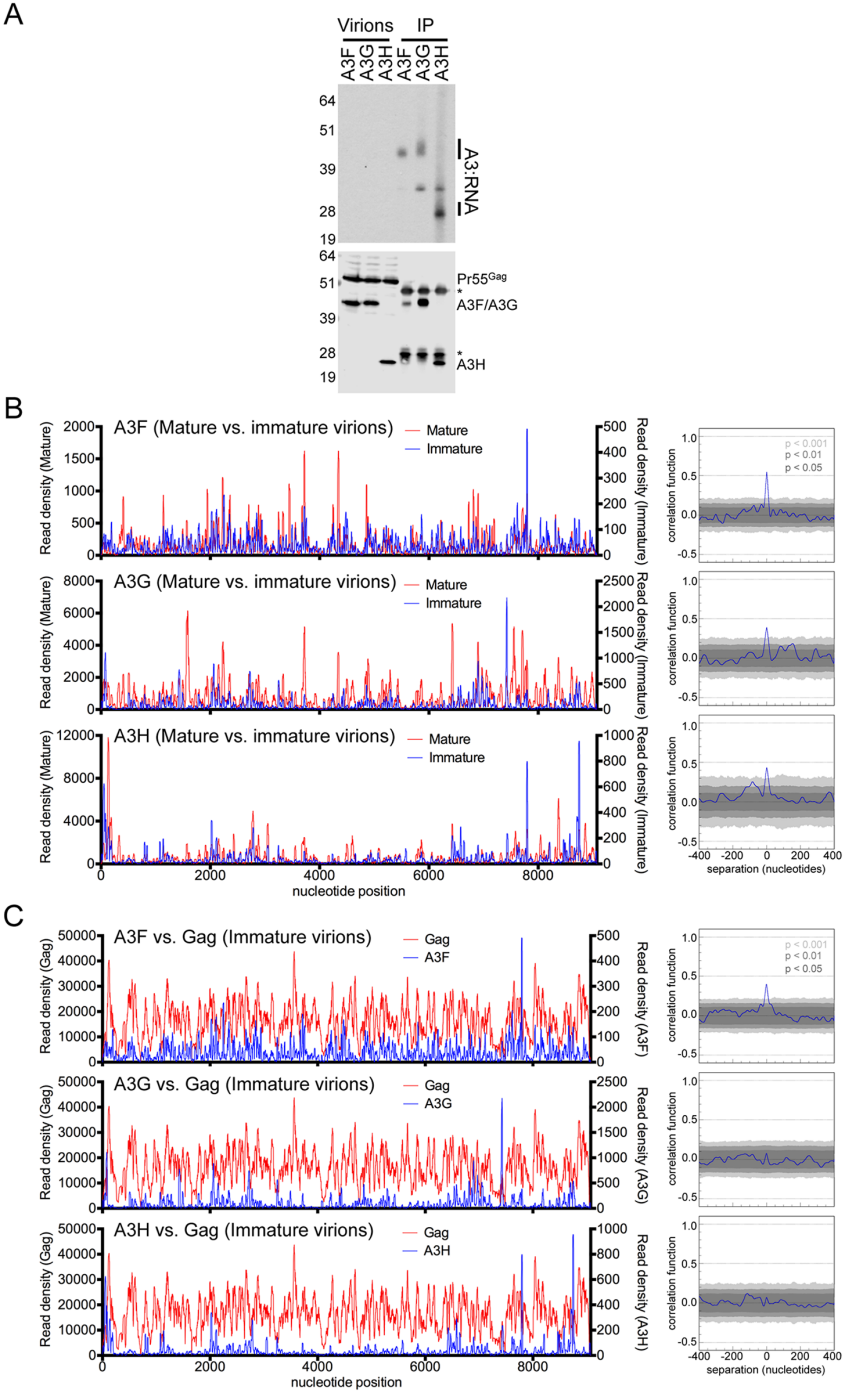
Interplay between A3 and Gag RNA binding to HIV-1 RNA in immature virions. (A) A3-RNA cross-linked complexes were immunoprecipitated from purified immature (PR defective) HIV-1_NL4-3 ΔVif_ virions derived from proviral plasmid-transfected HEK 293T cells stably expressing 3×HA-tagged A3F, A3G or A3H proteins. Complexes were visualized by autoradiography (top) and simultaneous Western blot analyses using anti-HA and anti-Gag antibodies (bottom). Asterisks indicate the presence of IgG heavy and light bands in the immunopreciptated fractions. (B) Comparisons of read density frequency distributions on the HIV-1_NL4-3_ genome for CLIP experiments in which A3 was immunoprecipitated from purified mature or immature virions (left). Correlation analyses of A3 binding frequencies across the viral RNA genome in mature and immature virions (right). (C) Comparisons of read density frequency distributions on the HIV-1_NL4-3_ genome for CLIP experiments in which A3 or Gag was immunoprecipitated from immature virions (left). Correlation analyses of A3 and Gag binding frequencies across the viral RNA genome in immature virions (right).

We noticed that the apparent preference of A3 proteins for G-rich and A-rich RNA sequences was reminiscent of the two modes of RNA binding exhibited by the HIV-1 Gag and NC proteins, before, during and after HIV-1 particle assembly [42]. Therefore, to investigate the relationship between A3 and Gag binding specificities, data obtained in A3 and Gag CLIP experiments [42] were subjected to correlation analysis (Figs 6 and 7, S9 and S10 Figs). The RNA binding profile of A3 proteins and Gag on the HIV-1 genome in infected cells exhibited no statistically significant correlation (Figs 6A and S9A). However, we noticed a marked reduction in A3 binding at sites of high Gag occupancy, including the 5' leader and Rev Response Element (RRE). This finding suggested that A3 might be occluded from these sites as a consequence of high Gag occupancy, or alternatively as a consequence of a high degree of RNA secondary structure at these sites. Although correlation between Gag and A3 binding to viral RNA in cells was not statistically significant, we did observe similar binding profiles in regions of the viral genome that are thought to have less secondary RNA structure (e.g. nucleotides 4000–6000, S9A Fig). The lack of overall statistical significance is likely be due to the fact that the dominant Gag binding signal to viral RNA in cells occurs in highly structured regions of RNA, including psi (Ψ) and the RRE while A3 has been reported to exhibit preference for single-stranded RNA [47].

We next compared the RNA binding profile of A3F, A3G and A3H to the HIV-1 genome with that of NC and Gag in mature and immature virions, respectively, using NC and Gag CLIP-seq data [42]. Remarkably, statistically significant and, in many areas, visually obvious correlation between A3-RNA and NC-RNA binding was observed in mature virions (Figs 6B, S9B). This was especially true for A3G and A3H, indicating that A3 and NC proteins have a similar RNA binding specificity in mature HIV-1 virions. In immature (protease deficient) virions, A3 proteins exhibited a similar pattern of binding to viral RNA as they did mature virions, albeit with some regions in which clear differences were apparent (Fig 7A and 7B, S10A Fig). The discrepancies suggest that some degree of change in A3 RNA binding specificity or target RNA availability occurs during virion maturation. Curiously, A3F RNA binding exhibited higher degree of correlation in mature versus immature virions than did A3G- and A3H-RNA binding (Fig 7B, S10A Fig).

In immature virions, the patterns of A3G and A3H protein binding to the viral genome did not correlate with the pattern of Gag binding (Fig 7C and S10B Fig), in contrast to the situation in mature virions (Figs 6B, S9B). However, binding of A3F and Gag to the viral genome in immature virions were weakly correlated (Fig 7C and S10B Fig). While the intrinsic RNA binding specificity of A3 proteins is not expected to change during virion genesis, the manner in which Gag competes with A3 proteins for sites on the viral RNA is expected to change, as Gag-RNA binding specificity changes dramatically during virion genesis [42]. These findings suggest that A3F better mimics the RNA binding specificity of HIV-1 Gag in immature virions, or is better able to compete with Gag for RNA binding sites than are A3G or A3H.

### Comparative analysis of A3, Gag and NC binding to cellular 7SL RNA during HIV-1 assembly

**Fig 8:**
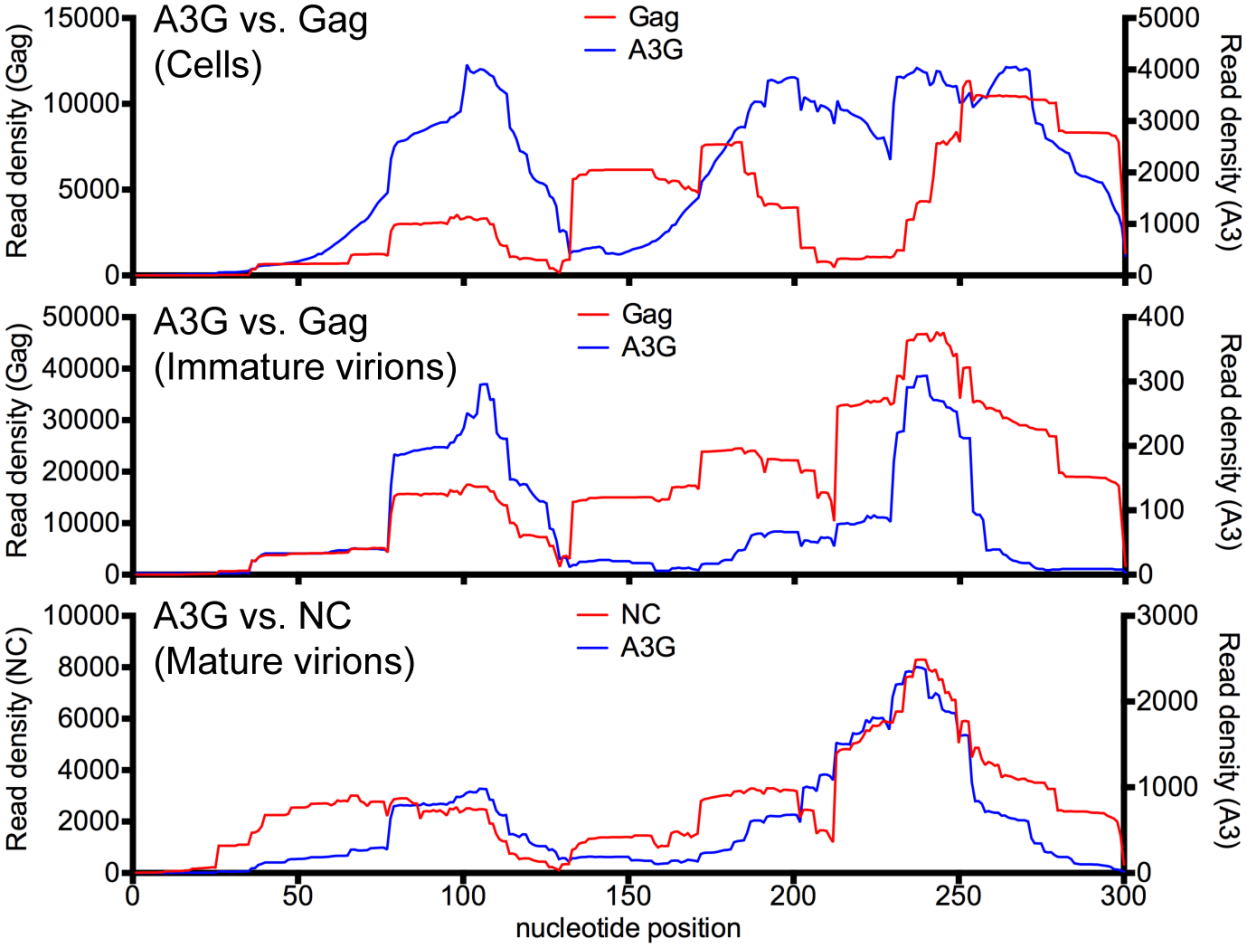
7SL1 RNA binding by A3, Gag and NC in cells, immature virions and mature virions. Comparisons of read density frequency distributions on the 7SL1 RNA for CLIP experiments in which A3G, Gag or NC were immunoprecipitated from cells, immature virions and mature virions respectively.

The aforementioned observations indicated that some degree of change in the RNA binding profile of A3 protein to viral RNA occurs during virion genesis, likely as a consequence of RNA representation, and competition with immature Gag during viron genesis. Ultimately, however, the A3 proteins and NC demonstrate an apparently similar viral RNA binding specificity in mature virions. To investigate whether these changes occurred with a different RNA target, we examined 7SL1, a cellular RNA that is ordinarily part of the SRP ribonucleoprotein complex but is highly enriched in the virions, through binding the HIV-1 Gag and NC proteins. 7SL is also bound by A3G and A3H proteins in infected cells and in virions [32–35, 48]. Specifically, we compared the A3G read densities with Gag and NC read densities on the 7SL1 RNA in infected cells, immature virions and mature virions, respectively (Fig 8). In cells, there was little correlation between A3 and Gag binding on the 7SL1 RNA. Indeed, A3 binding was observed in regions that had relatively few Gag binding events and *vice versa*. Strikingly, however, as Gag assembled into immature and then mature virions there was a progressive harmonization of binding patterns, and there was clear correlation between A3G and Gag or NC RNA binding in both immature and particularly mature virions. This finding reinforces the notion that A3 and HIV-1 NC proteins have apparently similar RNA binding specificities, using 2 different target RNAs, and suggests that competition by other proteins (including perhaps SRP proteins that bind 7SL proteins in cells) may affect RNA target availability and the apparent propensity of A3 proteins to bind to particular RNA elements in cells.

## Discussion

The RNA binding activity of A3 proteins is essential for incorporation into HIV-1 virions and restriction of virus replication. Our data confirm previous studies in that A3 proteins are promiscuous in the RNA molecules that they bind. Importantly however, our findings also argue that A3 proteins exhibit significant RNA binding selectivity that could facilitate their incorporation into nascent HIV-1 virions and thus contribute to their antiviral activity.

The majority of molecules that are bound by A3 proteins in cells were diverse messenger RNAs, while other cellular RNAs including 7SL RNA, miRNA, tRNA, rRNA were bound less frequently. The promiscuity of A3 protein binding is consistent with previous observations that A3G forms high molecular mass ribonucleoprotein complexes in cells that contain numerous cellular RNAs and RNA binding proteins [49] including poly(A)-binding proteins (PABPs), YB-1, RNA helicases, ribosomal proteins, and Staufen1 [50]. A3G has been found to localize to mRNA processing (P) bodies, cytoplasmic compartments that are responsible for the degradation and storage of non-translating messenger RNAs [51]. Although P-body-association does not appear to be necessary for antiviral activity [52, 53], RNA binding could obviously influence, or be influenced by, localization to these RNA-rich cellular structures.

Although RNA binding by A3 proteins is promiscuous, our data show that A3 proteins preferentially bind HIV-1 viral RNA in infected cells. Moreover, A3F, A3G and A3H proteins bound, with similar, reproducible, non-uniform distributions along the length of the HIV-1 genome. The RNA sequences that are preferentially bound by A3 proteins, and regions of the viral genome that are preferentially targeted could be governed by several factors including: (i) RNA abundance (ii) RNA binding specificity inherent to the A3 proteins (iii) secondary RNA structures present in RNAs, that might differ in cells and in virions and (iv) competition by other RNA binding proteins (most notably Gag). Like all lentiviruses, HIV-1 genomic RNA has a characteristic A-rich nucleotide composition, and also exhibits secondary structure, with regions that have greater or lesser propensity to exhibit single-stranded and double-stranded character [54]. HIV-1 RNA also associates intimately with Gag during virion genesis. Overall, our data suggests that each of the above factors impacts how A3 proteins bind to viral RNA prior to, during and after virion encapsidation.

Analysis of A3 binding to a complex RNA source (cellular mRNA) revealed that A3F, A3G and A3H each preferred to bind to G-rich or A-rich sites, and motif analysis confirmed this evident G/A-preference. This finding is consistent with a previous study which found that A3-NC complex formation could be driven *in vitro* by single-stranded RNA molecules that contain G residues, but not single-stranded RNA molecules that lacked G-bases (note that NC is also selective for G-bases) [47]. This modest level of selectivity likely underlies A3 binding to diverse RNA molecules, and would create a profound barrier to the selection of A3-resistant viral strains through changes in A3 binding sites on viral RNA. However, this level of selectivity would also reduce the number of ‘distracting’ RNA binding sites in cellular RNAs that are sampled by A3 proteins, and thereby potentially increase the efficiency with which A3 proteins are incorporated into virions, as compared to a completely nonspecific RNA binding protein.

Previous work has indicated that A3G binds specifically *in vitro* to single-stranded but not double-stranded RNA [47]. Thus, the accessibility of single stranded RNA would likely affect the pattern of HIV-1 genome binding. Indeed, our CLIP data show that the Rev Response Element (RRE) is disfavored for binding by each human A3 protein examined in this study and binding to the 5' leader was disfavored by A3F and A3G. However, A3H exhibited significant binding to the 5' leader, suggesting that RNA binding specificity is not determined by the presence or absence of RNA structure alone in all cases.

We previously reported that the viral Gag and NC proteins, like A3 proteins, bind to G- and A-rich rich sequences at different stages of virion genesis [42]. Nevertheless, an unexpected finding was that A3 proteins and NC targeted similar sites in the HIV-1 genome in mature virions. These findings suggest that A3 has evolved to partially mimic the RNA binding specificity of the HIV-1 NC domain of Gag, with NC targeting G-rich and A-rich sequences in order to ensure RNA packaging and A3 proteins targeting RNA in a similar manner to drive its own efficient incorporation into nascent virions. The apparently similar RNA binding specificity exhibited by HIV-1 NC and A3 proteins for viral RNA sequences was also evident with 7SL1 RNA, which is abundantly incorporated into virions. However, we also noted that the pattern of A3 binding to both viral and 7SL1 RNAs changed somewhat as virions assembled and matured. One likely explanation for this is the particularly intimate RNA association of the immature Gag protein, transiently, during virion genesis. Perhaps some of the preferred binding sites of the A3 proteins are occluded by Gag during immature virion assembly. Indeed, it is conceivable the RNA occlusion could be a countermeasure against A3 proteins that might be employed by retroviruses that lack a Vif protein.

Overall, this study suggests a model whereby A3 protein encapsidation into virions is facilitated by an RNA binding preference that has been acquired through natural selection so as to strike a balance between selectivity and promiscuity. Thus, only a subset of possible RNA binding sites, that are loosely defined by their nucleotide composition and enriched in lentiviral RNAs, are sampled by A3F, A3G and A3H proteins. Thus, many potentially distracting RNA binding sites are avoided. Conversely, A3F-, A3G- and A3H-RNA binding appears sufficiently non-selective so as to render the evolution of viral escape mutants whose RNA genome does not bind these proteins near impossible, and may thus have driven the acquisition of the *vif* gene for antagonism of A3 proteins.

## Materials and Methods

### Plasmids

The pLHCX-3×HA-A3F and pLHCX-3×HA-A3G plasmids were constructed by PCR-amplification of the open reading frames from pCR3.1 expression vectors encoding A3F and A3G with primers that contain 5' and 3' NotI and XhoI sites respectively. Amplicons were inserted into a pCR3.1 expression vector containing sequence encoding a 3×HA-tag 5' to the NotI site. The 3×HA-A3F and 3×HA-A3G cassettes were then subcloned into pLHCX using SnaBI and HpaI sites. The pLHCX-A3H-3×HA plasmid was generated by PCR-amplification of the open reading frame from a pTR600-A3H-3×HA plasmid [55] with primers that contain terminal HindIII sites for digestion and ligation into the pLHCX vector. HIV-1_NL4-3 ΔVif_ has been previously described [56]. HIV-1_NL4-3 ΔVif_/PR^−^was constructed by ligating an AgeI/SpeI fragment encoding an inactive PR protein of a previously described HIV_NL4-3_/PR^−^plasmid [57] into HIV-1_NL4-3 ΔVif_.

### Cells and viruses

Human Embryonic Kidney (HEK) 293T cells and MT4 cells (American Type Culture Collection) were maintained in Dulbecco’s Modified Eagle Medium (DMEM; Corning) or Roswell Park Memorial Institute medium (RPMI; Corning), respectively, supplemented with 10% fetal calf serum (FCS; Corning) and gentamicin. HEK 293T cells and MT4 cells that stably express 3×HA-tagged A3 proteins were produced by transduction of cells with MLV-based retroviral vectors. To generate the MLV-based retroviral vectors, approximately 5 ×10^5^ HEK 293T cells were co-transfected using polyethyleneimine (PolySciences) with 200 ng of a vesicular stomatitis virus G protein (VSV-G) expression plasmid, 1 μg of a MLV GagPol expression plasmid, and 1 μg of a 3×HA-tagged protein-expressing MLV vector. At 48 h post-transfection, cell culture supernatants containing MLV-based retroviral vectors were harvested, filtered and 200 μl was used to transduce approximately 5 ×10^5^ cells in the presence of 5 μg/ml polybrene (Hexadimethrine bromide; Sigma). At 48 h post-transduction, cells were selected in 50 μg/ml hygromycin-B (Corning). Single cell clones were derived by limiting dilution, picked and recultured, and protein expression was confirmed by Western blotting. The HIV-1_NL4-3 ΔVif_ virus that was used to infect cells was prepared by co-transfecting approximately 8 ×10^6^ HEK 293T cells with 22 μg of the HIV-1_NL4-3 ΔVif_ plasmid and 5.5 μg of the VSV-G expression plasmid using polyethylenimine. At 48 h post-transfection cell culture supernatants containing virions were filtered and virus titers were determined on MT4-LTR-GFP indicator cells using FACS analysis.

### Antibodies

The mouse monoclonal anti-HA (HA.11 Covance) was used in CLIP assays. The rabbit polyclonal anti-HA (600-401-384 Rockland) and the mouse monoclonal anti-HIV-1 p24CA (183-H12-5C, NIH AIDS Reagent Program) were used in Western blot analyses.

### CLIP-seq

The CLIP method that was used in this study has been described previously [42] and was adapted from previously reported HITS-CLIP and PAR-CLIP protocols [43, 44], and is described briefly here.

#### Infection and transfection

For CLIP experiments, approximately 1 ×10^7^ HEK 293T or MT4 cells that stably express 3×HA-tagged A3 proteins were either infected with HIV-1_NL4-3 ΔVif_ at an MOI of 2 in the presence of 5 μg/ml polybrene or, for HEK 293T cells, transfected with 25 μg of HIV-1_NL4-3 ΔVif_/PR^−^proviral plasmid using polyethylenimine. Approximately 14 h prior to harvesting cells and virions, 100 μM 4-thiouridine (4SU; Sigma) or 6-thioguanosine (6SG; Sigma) was added to the cell culture medium.

#### Cross-linking, lysis and nuclease treatment

Cells were briefly rinsed with ice cold PBS and irradiated once for HEK 293T cells, or twice for MT4 cells, with 0.15 J/cm^2^ UV (λ = 365 nm) in a Stratalinker 2400 UV crosslinker (Stratagene). Cell culture supernatants containing virions were harvested and filtered with a 0.22 μm Steriflip filter unit (Millipore) and virions were purified through a 20% sucrose cushion in PBS by ultracentrifugation at 96,467 ×*g* (avg) in a SW28 rotor for 2 h, 4°C. Virus pellets were resuspended in 500 μl PBS and irradiated twice with 0.15 J/cm^2^ UV (λ = 365 nm). Cells and virions were lysed in NP-40 lysis buffer (50 mM HEPES [pH 7.5], 150 mM KCl, 2 mM EDTA, 0.5% [v/v] NP-40, 1 mM dithiothreitol [DTT], 1× EDTA-free protease inhibitor cocktail [Roche]). After 30 min lysis on ice, the soluble fraction was separated by centrifugation at 17,000 × *g* for 5 min, 4°C. For lysates that contained A3F-3×HA the soluble lysate was adjusted to 1% NP-40, 0.25% Na-deoxycholate (Sigma) and 1% SDS and incubated for 15 min at 4°C, then the lysate was diluted 1:5 with dilution buffer (50 mM HEPES [pH 7.5], 150 mM KCl, 2 mM EDTA, 1 mM dithiothreitol [DTT], 1× EDTA-free protease inhibitor cocktail [Roche]). Lysates were subsequently treated with 20 U/ml of RNase A (Fermentas) and 60 U/ml with DNaseI (Roche) for 5 min at 37°C and then transferred to ice.

#### Immunoprecipitation, 3' dephosphorylation and 5' ATP-(γ-^32^P) labeling

Protein G-conjugated magnetic Dynabeads (Invitrogen) were washed twice with and resuspended in two bead volumes of citrate-phosphate buffer (4.7 g/L citric acid, 9.2 g/L Na_2_HPO_4_ [pH 5.0]). The beads were then incubated with 50 μg/ml of a mouse monoclonal anti-HA for 30 min, room temperature prior to being washed twice with 1 ml of citrate-phosphate buffer and resuspended in 1 bead volume. Approximately 20 μl/ml of beads was added to lysates, and then binding was performed for 2 h at 4°C in 1.5 ml siliconized low retention tubes (Fisher). After binding the beads were washed thoroughly twice with 1 ml of each of the following buffers: IP wash buffer (50 mM HEPES-KOH [pH 7.5], 300 mM KCl, 0.05% [v/v] NP-40, 0.5 mM DTT), LiCl buffer (250 mM LiCl, 10 mM Tris [pH 8.0], 1 mM EDTA, 0.5% NP-40, 0.5% Na-deoxycholate), NaCl buffer (50 mM Tris pH [7.4], 1 M NaCl, 1 mM EDTA, 0.1% SDS, 0.5% Na-deoxycholate, 1% NP-40), KCl buffer (50 mM HEPES-KOH [pH 7.5], 500 mM KCl, 0.05% [v/v] NP-40, 0.5 mM DTT), dephosphorylation buffer (50 mM Tris-HCl [pH 7.9], 100 mM NaCl, 10 mM MgCl_2_, 1 mM DTT). Thereafter, dephosphorylation was performed in 1 bead volume of dephosphorylation buffer (50 mM Tris-HCl [pH 7.9], 100 mM NaCl, 10 mM MgCl_2_, 1 mM DTT) containing 0.5 U/μl calf-intestinal phosphatase (NEB) for 15 min at 37°C in a Thermomixer. The beads were then washed twice with 1 ml of each of phosphatase wash buffer (50 mM Tris-HCl [pH 7.5], 20 mM EGTA, 0.5% [v/v] NP-40) and polynucleotide kinase (PNK) Buffer (50 mM Tris-HCl [pH 7.5], 50 mM NaCl, 10 mM MgCl_2_, 5 mM DTT). The beads were subsequently resuspended in 1 bead volume of PNK buffer containing 0.5 μCi/μl γ-32P[ATP] ATP and 1 U/μl T4 PNK (NEB) and incubated for 30 min at 37°C in a Thermomixer. Thereafter, cold ATP was added to a final concentration of 20 μM and the incubation was continued for 10 min at 37°C. The beads were then washed once with 1 ml of PNK, LiCl, KCl and NaCl buffers. Beads were then resuspended in 50 μl of NuPAGE SDS-PAGE loading buffer and incubated at 72°C for 10 min (with constant mixing) to elute protein-RNA adducts.

#### RNA isolation, adapter ligations, reverse transcription and PCR

Protein-RNA adducts were separated by SDS-PAGE, transfered to nitrocellulose and detected by autoradiography. RNA was isolated by Proteinase K treatment and phenol:chloroform isolation as previously described [42]. Sequential 3' and 5' adapter ligations were then performed on the isolated RNA as previously described [42] resulting in RNA of unknown sequence that was flanked by known sequence that contained primer-binding sites for subsequent reverse transcription and PCR-amplification of the cDNA library as previously described [42]. Sequencing of the cDNA library was performed on an Illumina HiSeq 2000 platform.

### Bioinformatic and correlation analyses

The analysis pipeline that was used in this study has been previously described [42]. Briefly, the FASTX toolkit (http://hannonlab.cshl.edu/fastx_toolkit/) was used to process the raw reads prior to mapping. Reads that were fewer than 15 nt, did not contain the 3' adapter sequence or contained ambiguous nucleotides were excluded from further analyses. Reads were subsequently aligned using Bowtie [58] to the human genome (hg19) concatenated with the HIV-1_NL4-3_ genome, or to the viral genome alone, allowing for a maximum of 2 mismatches Reads derived from the R region of the HIV-1_NL4-3_ LTR have been displayed at the 5' end of the genome in our analyses and interpreted with caution. SAMtools [59] was used to generate pileups of the mapped reads which further analysed using in-house scripts. Cluster analysis was performed using PARalyzer [45] using parameters as previously described [42] and the generated clusters were annotated using in-house scripts according to the EMSEMBL v72 database [60]. PARalyzer-generated clusters were then used as input for the cERMIT motif finding tool [46]. Previously described PERL scripts [42] were used for determining nucleotide composition of clusters. Correlation analysis was examined by means of a correlation function (CF), the determination of which has been previously described in detail [42]. Briefly, the existence of a significant level of correlation between read density frequencies in two different data sets was examined by determining a CF. Given two data series, the CF for a certain separation of nucleotides (s) can be defined. High values of CF at s = 0 indicate that the locations and relative peak heights of the binding sites in the two data samples are correlated. Data sets derived from APOBEC3 CLIP-seq experiments in cells (not virions) show significantly positive values of the correlation function for a wide range of separations. This is due to these data sets having a bimodal read density, with all the peaks with high number of reads located in one half of the data array (i.e. the 3' end of the genome). This bimodal data structure introduces a structure in the correlation function, with systematically positive values for s < l and negative values for s > l, where l is the extent of the region that concentrates all high amplitude peaks. We did not attempt to model this bimodal data structure when generating the confidence regions, which results in an overestimation of the significance of positive correlation values when these data sets were analyzed.

## Supporting Information

S1 Fig Viral RNA binding by A3F, A3G and A3H in cells and mature virions. Comparison of the read densities on the HIV-1_NL4-3_ genome obtained from two independent A3F, A3G and A3H CLIP experiments in infected cells and mature virions. Read densities for the 5' 2000 nucleotides of the viral genome are shown for clarity.
(TIF)

S2 Fig Comparison of viral RNA binding by A3F and A3G in HEK 293T and MT4 cell CLIP experiments. Frequency distributions of read densities on the HIV-1_NL4-3_ genome in A3F- and A3G-CLIP experiments done using infected 293T cells or MT4 cells (top). Data from MT4 cell CLIP experiments using a single cell clone for A3F and two single cell clones for A3G are shown. An expanded view of the frequency distributions of read densities for the 5' 2000 nucleotides of the viral genome are also shown for clarity (bottom). Correlation analysis of A3 binding frequencies on the viral RNA genome HEK 293T and MT4 A3F- and A3G-CLIP experiments (right).
(TIF)

S3 Fig Comparison of viral RNA binding by A3G and A3H in 4SU- and 6SG-based CLIP experiments. (A) A3-RNA cross-linked complexes were immunoprecipitated from mock infected or HIV-1_NL4-3 ΔVif_ infected HEK 293T cells stably expressing 3×HA-tagged A3G or A3H proteins that had been supplemented with 6SG and UV-irradiated. Complexes were visualized by autoradiography (top) and Western blot analysis using an anti-HA antibody (bottom). (B) Frequency distributions of read densities on the HIV-1_NL4-3_ genome in 4SU- and 6SG-based A3G- and A3H-CLIP experiments (top). An expanded view of the frequency distributions of read densities for the 5' 2000 nucleotides of the viral genome are also shown for clarity (bottom). Correlation analysis of A3 binding frequencies on the viral RNA genome in 4SU- and 6SG-based A3G- and A3H-CLIP experiments (right). (C) Classification of individual reads that map to the human and HIV-1_NL4-3_ genomes from A3G and A3H CLIP experiments with uninfected and infected cells in 6SG-based CLIP experiments.
(TIF)

S4 Fig Comparison of A3F, A3G and A3H RNA binding in cells and mature virions. (A) Comparison of read density frequency distributions on the HIV-1_NL4-3_ genome for CLIP experiments in which the same A3 protein was immunoprecipitated from infected cells or mature virions. Read densities for the 5' 2000 nucleotides of the viral genome are shown for clarity. (B) Comparisons of read density frequency distributions on the HIV-1_NL4-3_ genome for CLIP experiments in which different A3 proteins were immunoprecipitated from infected cells or mature virions, as indicated. Read densities for the 5' 2000 nucleotides of the viral genome are shown for clarity.
(TIF)

S5 Fig Binding of A3 proteins to cellular mRNAs. Depiction of the proportions of reads that were derived from 5'UTR, 3'UTR and coding sequences (CDS), for reads that mapped to cellular mRNAs in A3-CLIP experiments. The total number of reads analyzed is indicated below each bar.
(TIF)

S6 Fig Binding of A3F to cellular mRNAs. Read density frequency distribution in CLIP and RNA-seq experiments for the 10 most frequently A3F-bound cellular mRNAs The 5'untranslated (UTR), coding sequence (CDS) and 3'UTR regions are indicated for the CLIP reads.
(TIF)

S7 Fig Binding of A3G to cellular mRNAs. Read density frequency distribution in CLIP and RNA-seq experiments for the 10 most frequently A3G-bound cellular mRNAs The 5'untranslated (UTR), coding sequence (CDS) and 3'UTR regions are indicated for the CLIP reads.
(TIF)

S8 Fig Binding of A3H to cellular mRNAs. Read density frequency distribution in CLIP and RNA-seq experiments for the 10 most frequently A3H-bound cellular mRNAs The 5'untranslated (UTR), coding sequence (CDS) and 3'UTR regions are indicated for the CLIP reads.
(TIF)

S9 Fig Interplay between A3, Gag and NC binding to the HIV-1 genome in infected cells or mature virions. (A) Comparisons of read density frequency distribution on the HIV-1_NL4-3_ genome for CLIP experiments in which A3 proteins or Gag were immunoprecipitated from infected cells. Read densities for nucleotides 2000–6000 of the viral genome are shown. (B) Comparisons of read density frequency distributions on the HIV-1_NL4-3_ genome for CLIP experiments in which A3 and NC were immunoprecipitated from purified mature virions. Read densities for nucleotides 2000–6000 of the viral genome are shown.
(TIF)

S10 Fig Interplay between A3 and Gag RNA binding to the HIV-1 genome in immature virions. (A) Comparisons of read density frequency distributions on the HIV-1_NL4-3_ genome for CLIP experiments in which A3 was immunoprecipitated from purified mature or immature virions. Read densities for the 5' 2000 nucleotides of the viral genome are shown for clarity. (B) Comparisons of read density frequency distributions on the HIV-1_NL4-3_ genome for CLIP experiments in which A3 or Gag was immunoprecipitated from immature virions. Read densities for the 5' 2000 nucleotides of the viral genome are shown.
(TIF)
