# Supplementary figures and images for: The RNA Binding Specificity of Human APOBEC3 Proteins Resembles That of HIV-1 Nucleocapsid
Source: PLoS Pathog. 2016 Aug 19;12(8):e1005833. doi: 10.1371/journal.ppat.1005833 (PMC4991800; doi:10.1371/journal.ppat.1005833)

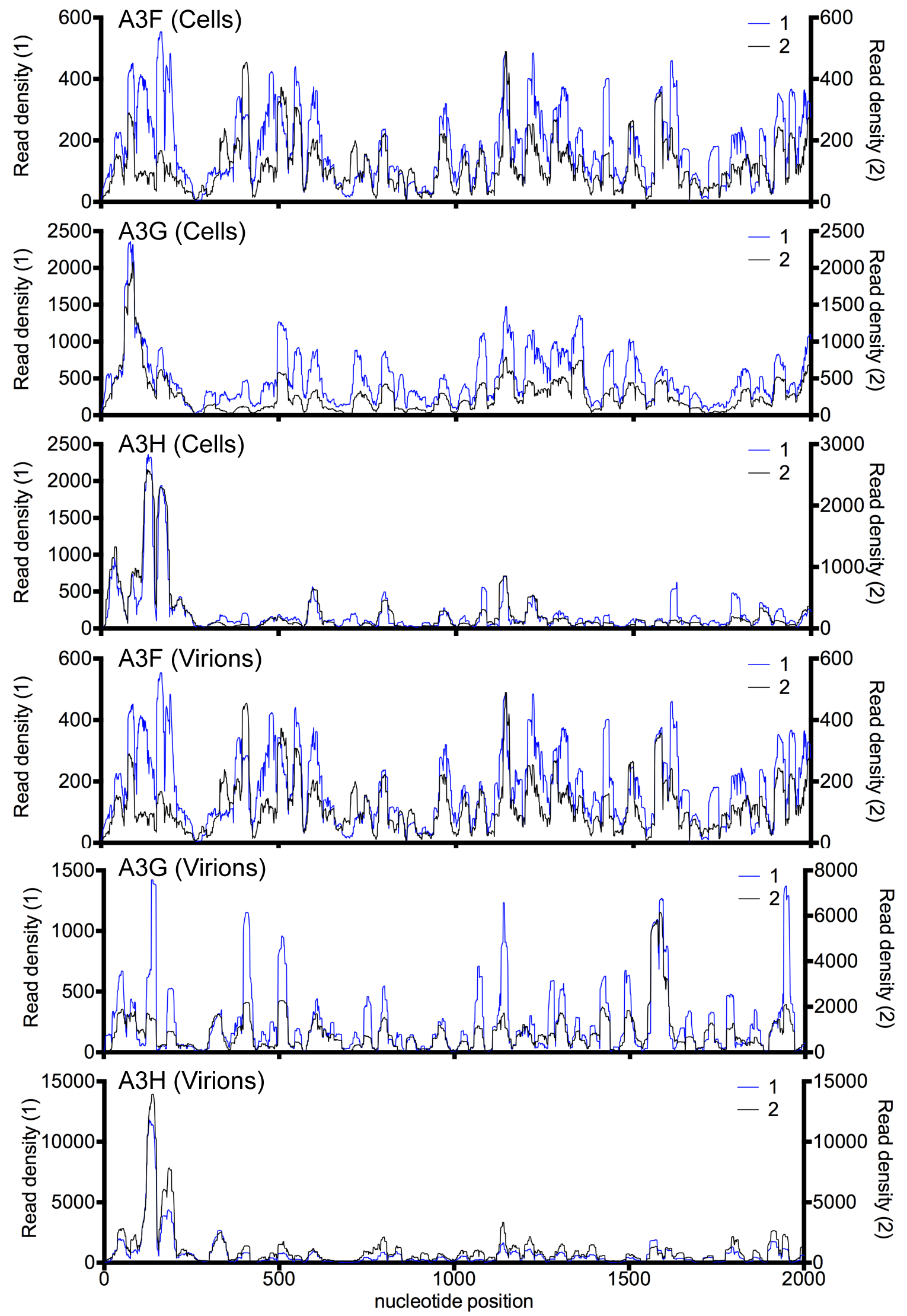

Supplement: S1 Fig — Comparison of the read densities on the HIV-1NL4-3 genome obtained from two independent A3F, A3G and A3H CLIP experiments in infected cells and mature virions. Read densities for the 5' 2000 nucleotides of the viral genome are shown for clarity. (TIF) [file ppat.1005833.s001.tif]

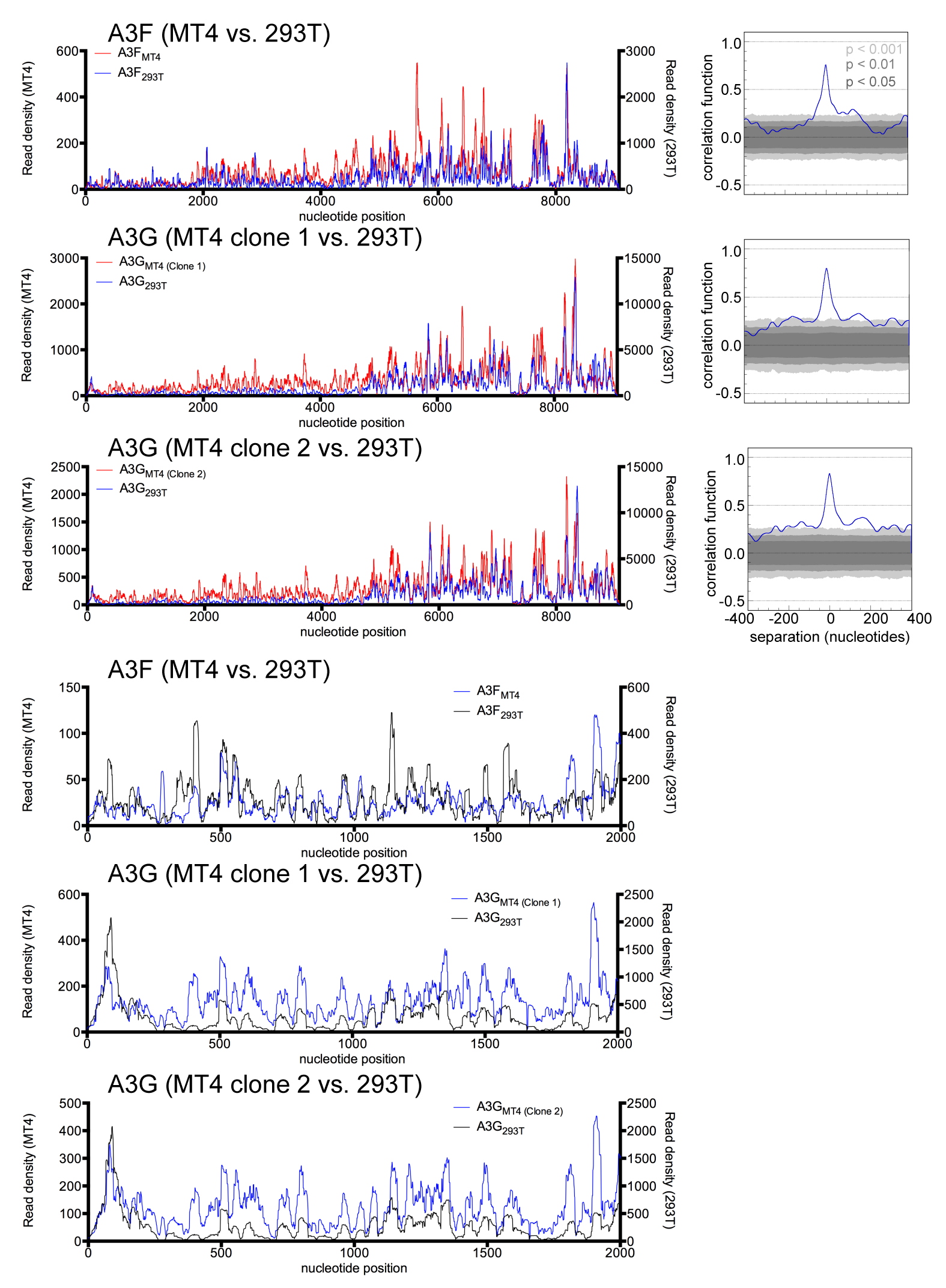

Supplement: S2 Fig — Frequency distributions of read densities on the HIV-1NL4-3 genome in A3F- and A3G-CLIP experiments done using infected 293T cells or MT4 cells (top). Data from MT4 cell CLIP experiments using a single cell clone for A3F and two single cell clones for A3G are shown. An expanded view of the frequency distributions of read densities for the 5' 2000 nucleotides of the viral genome are also shown for clarity (bottom). Correlation analysis of A3 binding frequencies on the viral RNA genome HEK 293T and MT4 A3F- and A3G-CLIP experiments (right). (TIF) [file ppat.1005833.s002.tif]

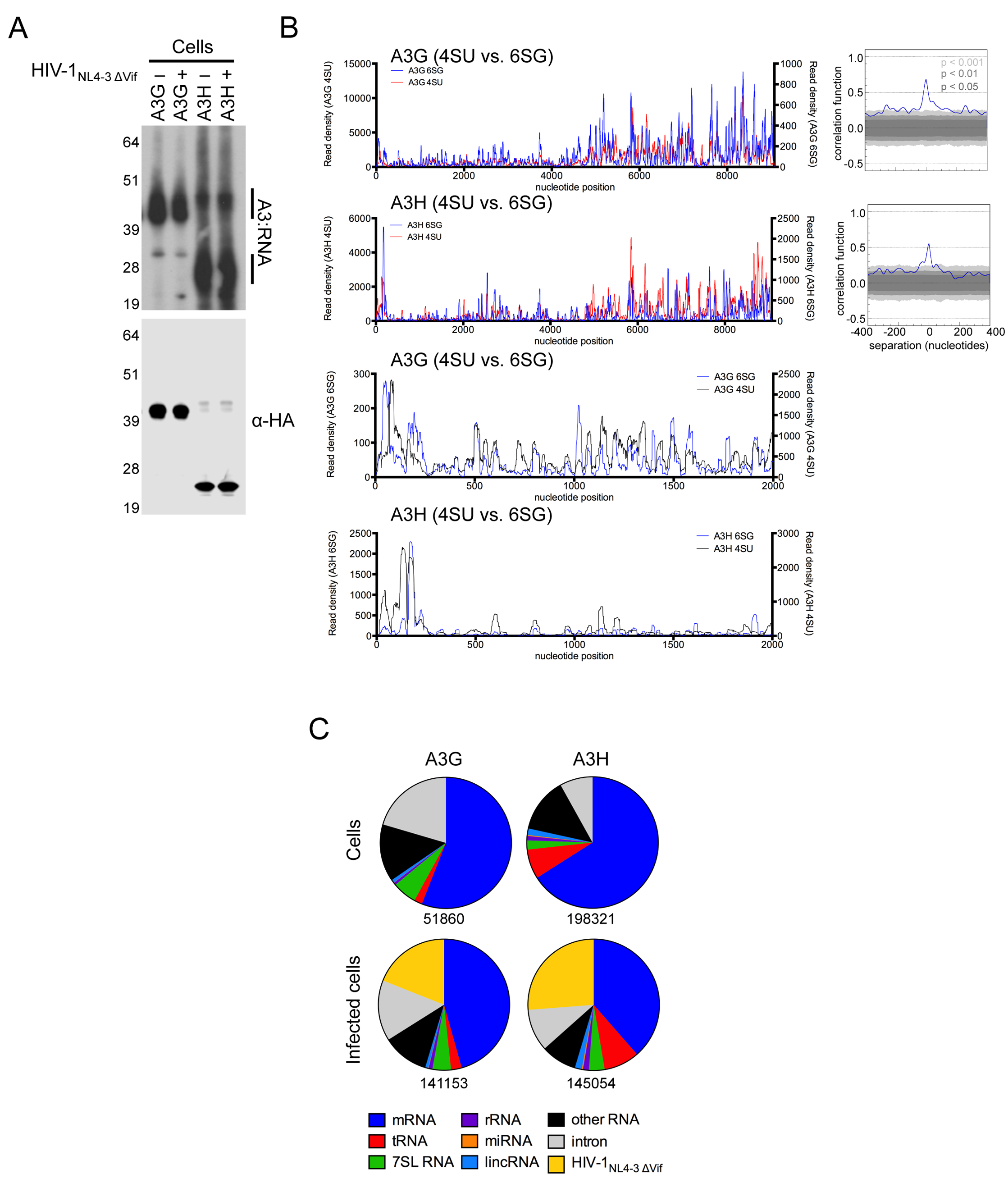

Supplement: S3 Fig — (A) A3-RNA cross-linked complexes were immunoprecipitated from mock infected or HIV-1NL4-3 ΔVif infected HEK 293T cells stably expressing 3×HA-tagged A3G or A3H proteins that had been supplemented with 6SG and UV-irradiated. Complexes were visualized by autoradiography (top) and Western blot analysis using an anti-HA antibody (bottom). (B) Frequency distributions of read densities on the HIV-1NL4-3 genome in 4SU- and 6SG-based A3G- and A3H-CLIP experiments (top). An expanded view of the frequency distributions of read densities for the 5' 2000 nucleotides of the viral genome are also shown for clarity (bottom). Correlation analysis of A3 binding frequencies on the viral RNA genome in 4SU- and 6SG-based A3G- and A3H-CLIP experiments (right). (C) Classification of individual reads that map to the human and HIV-1NL4-3 genomes from A3G and A3H CLIP experiments with uninfected and infected cells in 6SG-based CLIP experiments. (TIF) [file ppat.1005833.s003.tif]

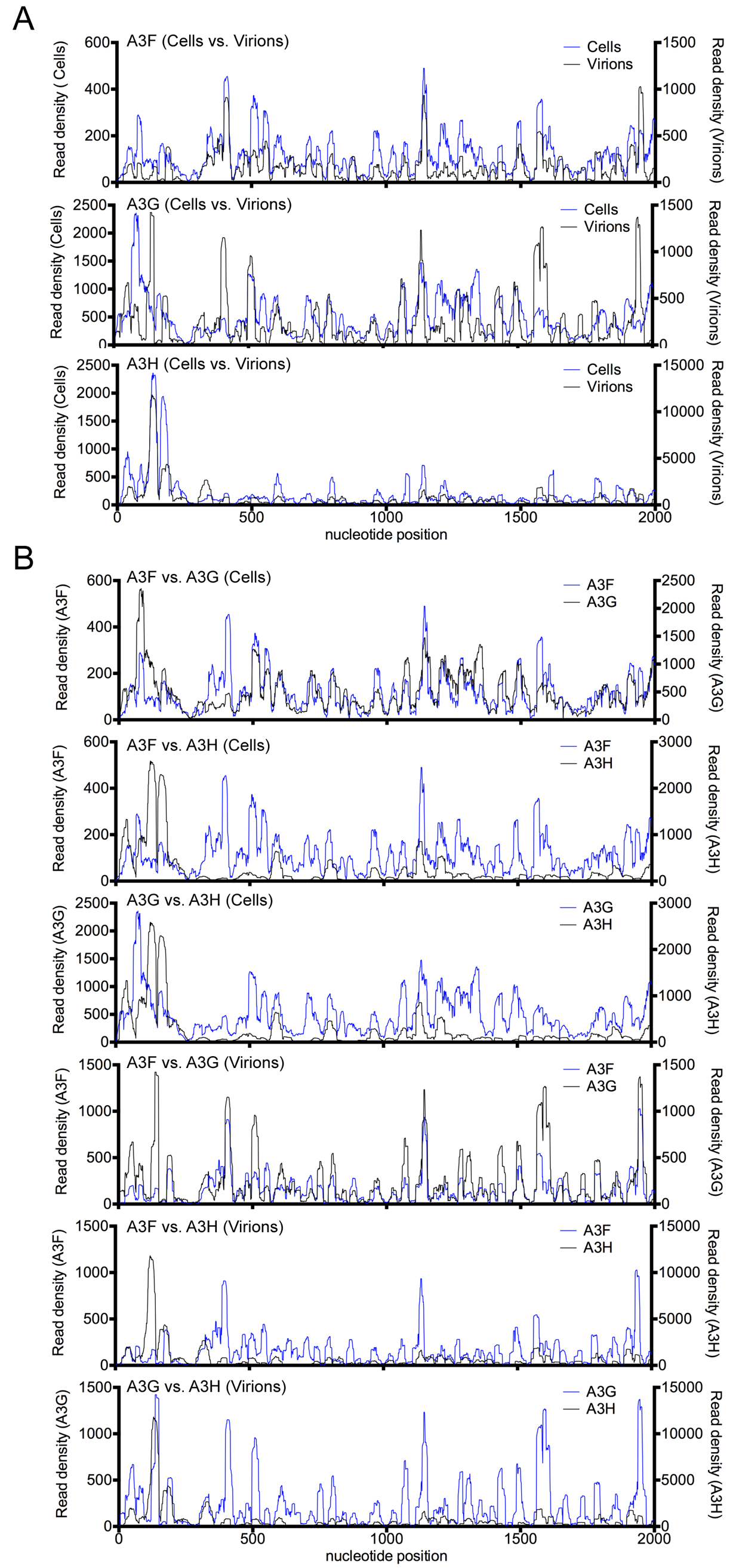

Supplement: S4 Fig — (A) Comparison of read density frequency distributions on the HIV-1NL4-3 genome for CLIP experiments in which the same A3 protein was immunoprecipitated from infected cells or mature virions. Read densities for the 5' 2000 nucleotides of the viral genome are shown for clarity. (B) Comparisons of read density frequency distributions on the HIV-1NL4-3 genome for CLIP experiments in which different A3 proteins were immunoprecipitated from infected cells or mature virions, as indicated. Read densities for the 5' 2000 nucleotides of the viral genome are shown for clarity. (TIF) [file ppat.1005833.s004.tif]

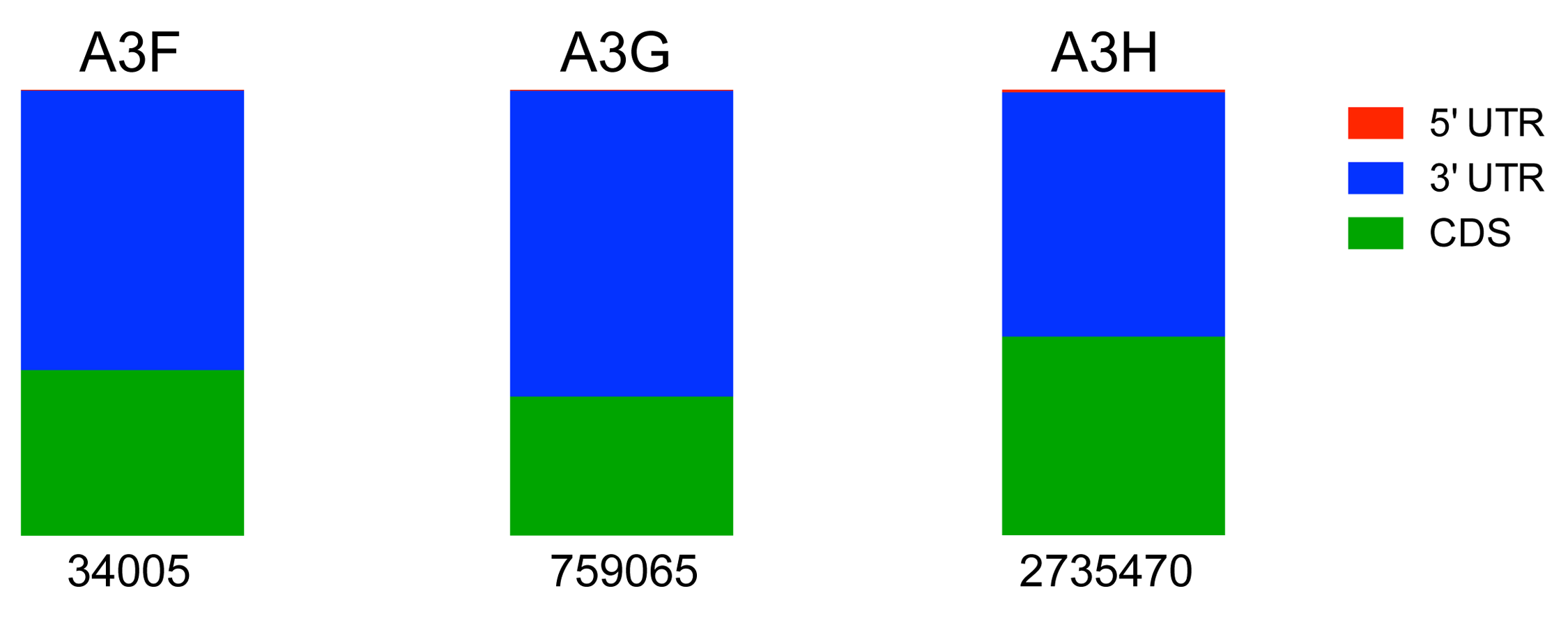

Supplement: S5 Fig — Depiction of the proportions of reads that were derived from 5'UTR, 3'UTR and coding sequences (CDS), for reads that mapped to cellular mRNAs in A3-CLIP experiments. The total number of reads analyzed is indicated below each bar. (TIF) [file ppat.1005833.s005.tif]

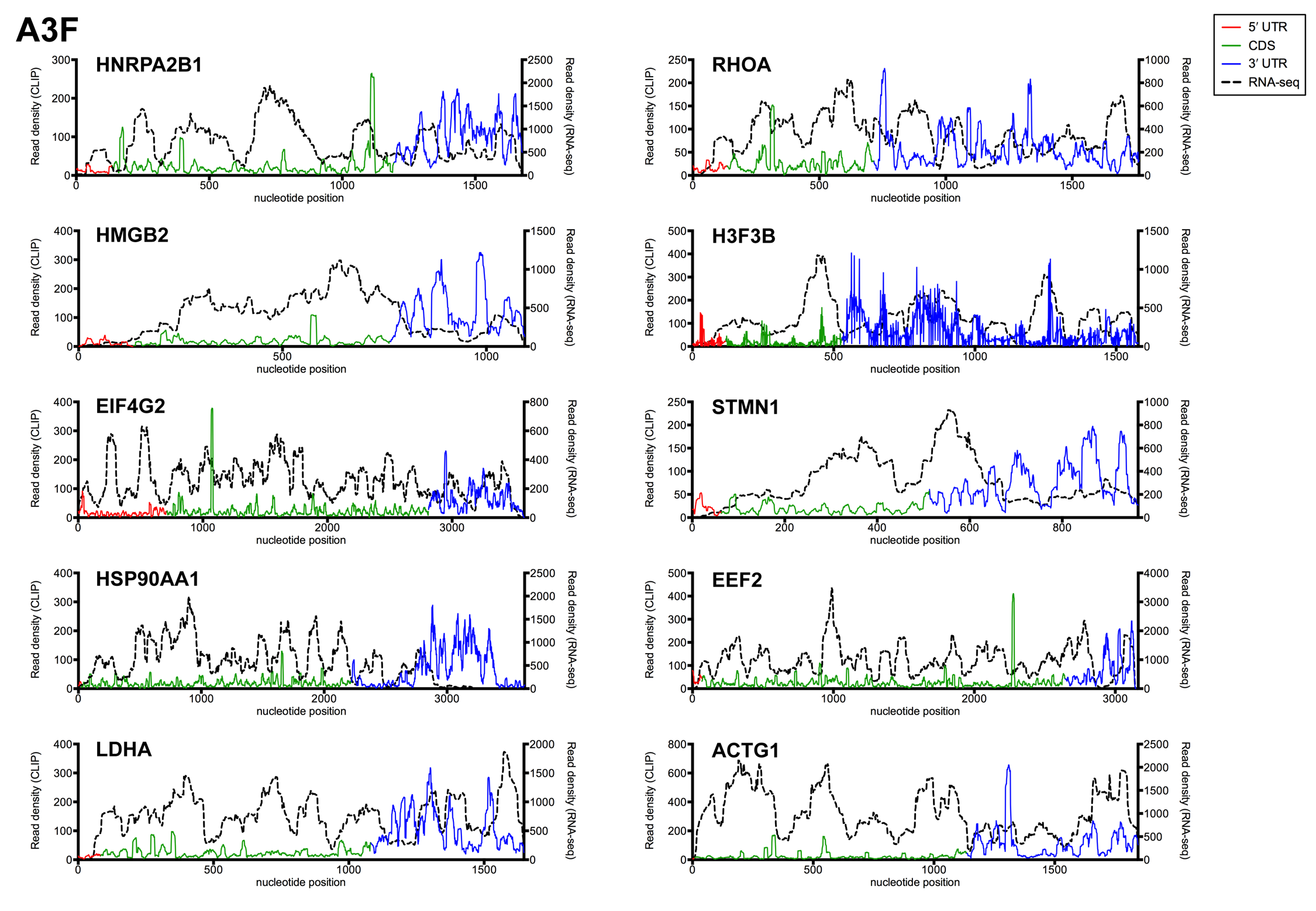

Supplement: S6 Fig — Read density frequency distribution in CLIP and RNA-seq experiments for the 10 most frequently A3F-bound cellular mRNAs The 5'untranslated (UTR), coding sequence (CDS) and 3'UTR regions are indicated for the CLIP reads. (TIF) [file ppat.1005833.s006.tif]

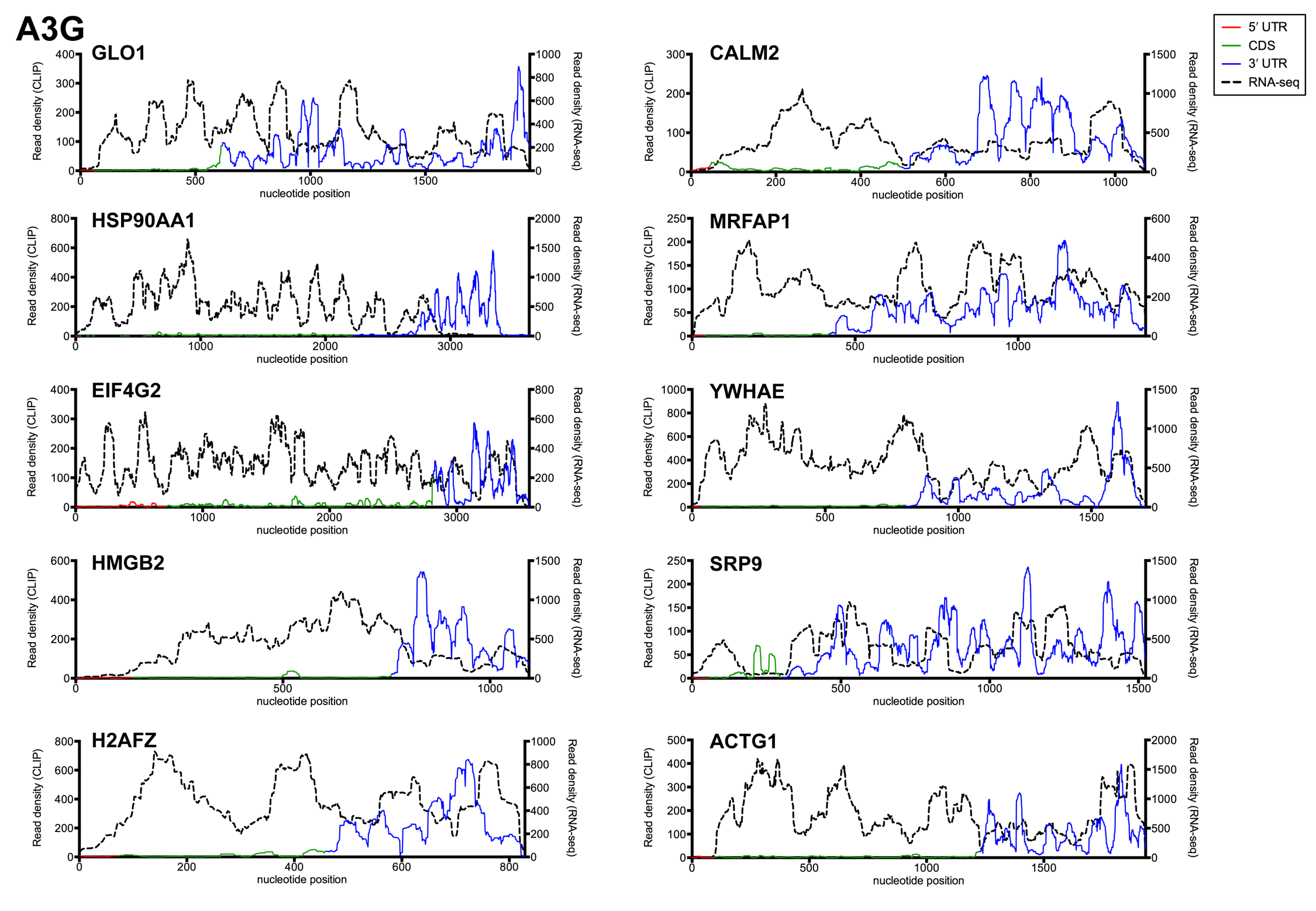

Supplement: S7 Fig — Read density frequency distribution in CLIP and RNA-seq experiments for the 10 most frequently A3G-bound cellular mRNAs The 5'untranslated (UTR), coding sequence (CDS) and 3'UTR regions are indicated for the CLIP reads. (TIF) [file ppat.1005833.s007.tif]

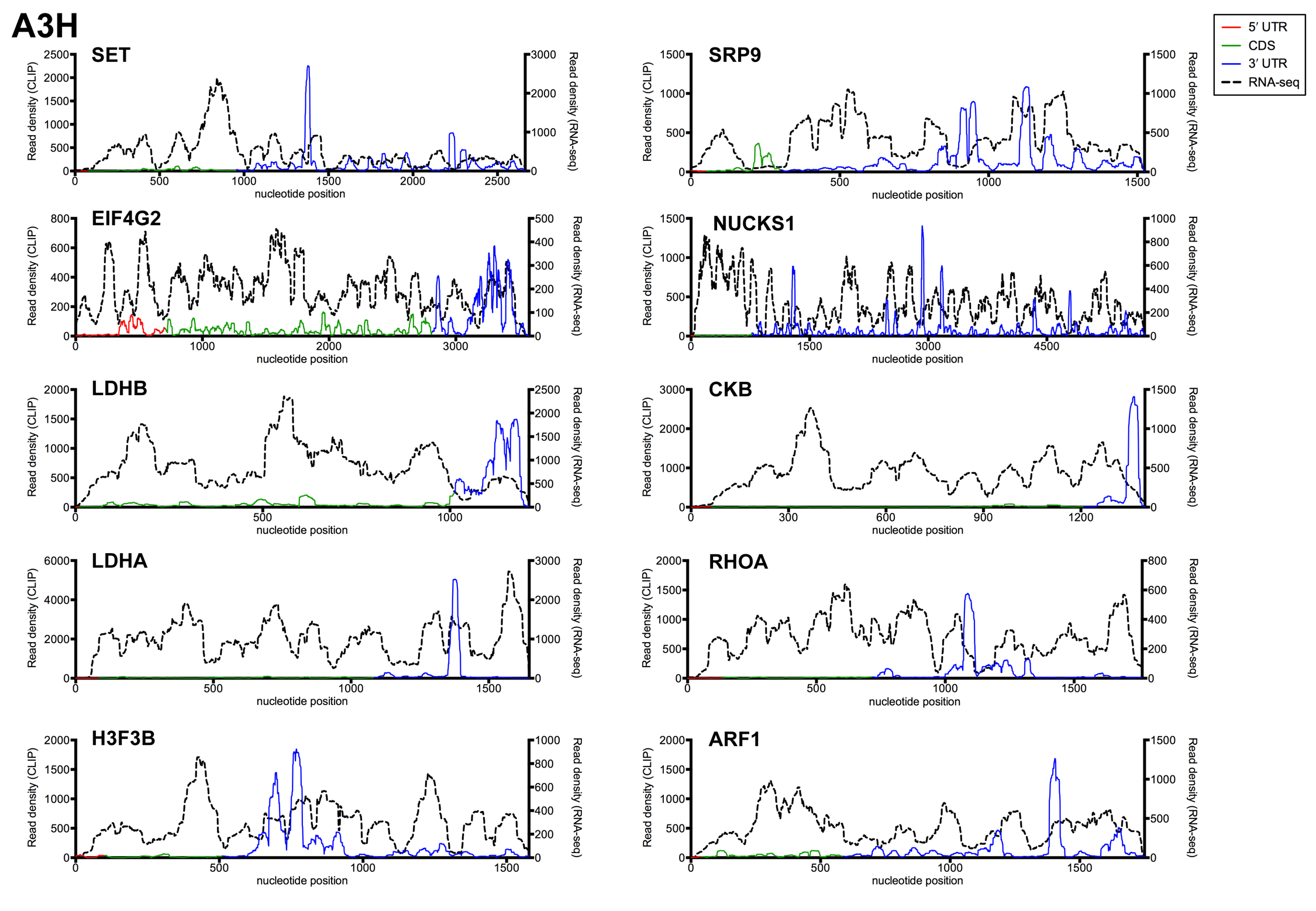

Supplement: S8 Fig — Read density frequency distribution in CLIP and RNA-seq experiments for the 10 most frequently A3H-bound cellular mRNAs The 5'untranslated (UTR), coding sequence (CDS) and 3'UTR regions are indicated for the CLIP reads. (TIF) [file ppat.1005833.s008.tif]

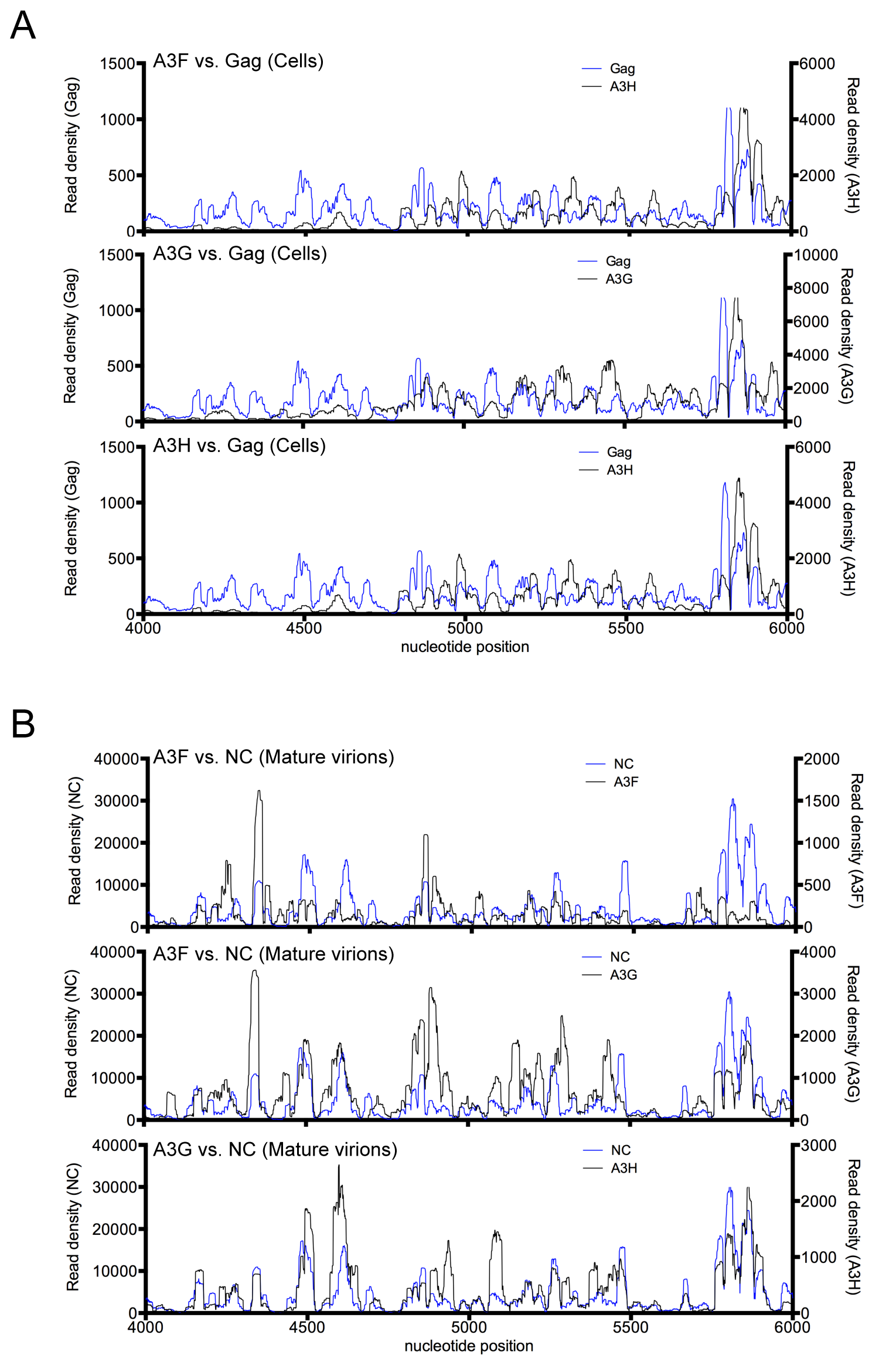

Supplement: S9 Fig — (A) Comparisons of read density frequency distribution on the HIV-1NL4-3 genome for CLIP experiments in which A3 proteins or Gag were immunoprecipitated from infected cells. Read densities for nucleotides 2000–6000 of the viral genome are shown. (B) Comparisons of read density frequency distributions on the HIV-1NL4-3 genome for CLIP experiments in which A3 and NC were immunoprecipitated from purified mature virions. Read densities for nucleotides 2000–6000 of the viral genome are shown. (TIF) [file ppat.1005833.s009.tif]

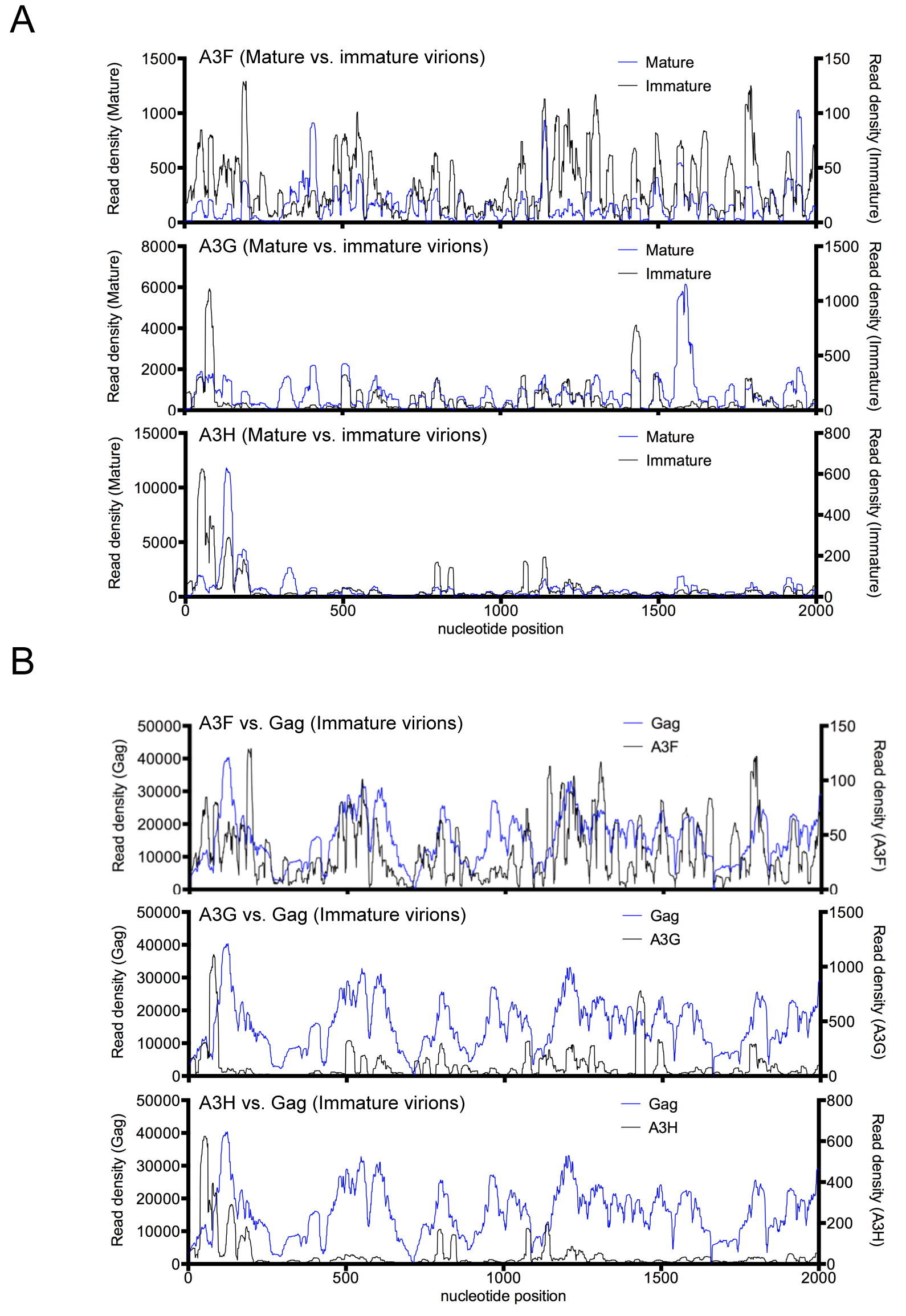

Supplement: S10 Fig — (A) Comparisons of read density frequency distributions on the HIV-1NL4-3 genome for CLIP experiments in which A3 was immunoprecipitated from purified mature or immature virions. Read densities for the 5' 2000 nucleotides of the viral genome are shown for clarity. (B) Comparisons of read density frequency distributions on the HIV-1NL4-3 genome for CLIP experiments in which A3 or Gag was immunoprecipitated from immature virions. Read densities for the 5' 2000 nucleotides of the viral genome are shown. (TIF) [file ppat.1005833.s010.tif]
